# Fides: Reliable trust-region optimization for parameter estimation of ordinary differential equation models

**DOI:** 10.1371/journal.pcbi.1010322

**Published:** 2022-07-13

**Authors:** Fabian Fröhlich, Peter K. Sorger

**Affiliations:** Laboratory of Systems Pharmacology and Department of Systems Biology, Harvard Medical School, Boston, Massachusetts, United States of America; Inria, FRANCE

## Abstract

Ordinary differential equation (ODE) models are widely used to study biochemical reactions in cellular networks since they effectively describe the temporal evolution of these networks using mass action kinetics. The parameters of these models are rarely known *a priori* and must instead be estimated by calibration using experimental data. Optimization-based calibration of ODE models on is often challenging, even for low-dimensional problems. Multiple hypotheses have been advanced to explain why biochemical model calibration is challenging, including non-identifiability of model parameters, but there are few comprehensive studies that test these hypotheses, likely because tools for performing such studies are also lacking. Nonetheless, reliable model calibration is essential for uncertainty analysis, model comparison, and biological interpretation.

We implemented an established trust-region method as a modular Python framework (fides) to enable systematic comparison of different approaches to ODE model calibration involving a variety of Hessian approximation schemes. We evaluated fides on a recently developed corpus of biologically realistic benchmark problems for which real experimental data are available. Unexpectedly, we observed high variability in optimizer performance among different implementations of the same mathematical instructions (algorithms). Analysis of possible sources of poor optimizer performance identified limitations in the widely used Gauss-Newton, BFGS and SR1 Hessian approximation schemes. We addressed these drawbacks with a novel hybrid Hessian approximation scheme that enhances optimizer performance and outperforms existing hybrid approaches. When applied to the corpus of test models, we found that fides was on average more reliable and efficient than existing methods using a variety of criteria. We expect fides to be broadly useful for ODE constrained optimization problems in biochemical models and to be a foundation for future methods development.

## Author summary

In cells, networks of biochemical reactions involving complex, time-dependent interactions among proteins and other biomolecules regulate diverse processes like signal transduction, cell division, and development. Precise understanding of the time-evolution of these networks requires the use of dynamical models, among which mass-action models based on ordinary differential equations are both powerful and tractable. However, for these models to capture the specifics of a particular cellular process, their parameters must be estimated by minimizing the difference between the simulation (of a dynamical variable such as a particular protein concentration) and experimental data (this is the process of model calibration). This is a difficult and computation-intensive process that has previously been tackled using a range of mathematical techniques whose strengths and weaknesses are not fully understood. In this manuscript, we describe a new software tool, fides, that makes rigorous comparison of calibration methods possible. Unexpectedly, we find that different software implementations of the same mathematical method vary in performance. Using fides, we analyze the causes of this variability, evaluate multiple improvements, and implement a set of generally useful methods and metrics for use in future modeling studies.

## 1 Introduction

Many cellular biochemical networks exhibit time-varying responses to external and internal stimuli. Modeling networks requires using dynamical models that capture key features of these networks at the level of individual bio-molecules but remain computationally tractable. Developing and testing these models requires time-resolved experimental data, but these datasets are usually severely limited, particularly for mammalian cells: only a subset of model species (e.g., proteins) are typically measured (observed), and these measurements are usually made at discrete timepoints. To partially compensate for the sparsity of measurements, the experimental system is typically observed under a range of conditions that differ in the strength of the stimulus and the presence or absence of inhibitory drugs and genetic mutations.

Given these challenges, mass-action biochemical systems have emerged as an effective means of modeling the temporal evolution of a wide range of cellular networks [1]. Mass action biochemistry is a continuum approximation (i.e., one in which a large number of well-mixed molecules are present in each reaction compartment) that can be modeled by ordinary differential equation (ODE) models. Although cells are not well-mixed systems, ODE modeling can be highly effective for describing biochemical processes in both eukaryotic and prokaryotic cells [2]. Few parameters in these models, e.g., the initial reactant concentrations and rate constants, are known a priori and must instead be estimated from the (often limited) data. Estimation is commonly formulated as an optimization problem, where the objective function describes the discrepancy between a given solution to the ODE and experimental data. Minimizing this discrepancy can be computationally demanding due to the numerical integration required when evaluating the objective function and its derivatives [3]. Moreover, optimization is complicated by the wide range of time scales and intrinsic non-identifiability of many biochemical models (a property related to their “sloppiness” [4]) and the structure of the experimental data.

Efficiently finding robust solutions to the optimization problem is essential for model analysis, including prediction of unseen conditions and attempts to understand the logic of the underlying biochemical system [5]. Optimized parameter values are often used to initialize model analysis, such as uncertainty quantification via the profile likelihood or sampling approaches [6, 7]. Similarly, parameter optimization is required when models are compared based on goodness of fit, using measures such as Akaike information criterion (AIC) or Bayesian information criterion (BIC), or when other complexity-penalizing methods are applied [8, 9].

In general, the optimization problem for ODE models is non-convex, resulting in few theoretical guarantees of convergence when numerical optimization is employed. It is therefore necessary to rely on empirical evidence to select appropriate optimization algorithms for any specific class of problems [3, 10]. With respect to optimization in general, parameter estimation for ODE-based biochemical models belongs to an uncommon class of problems having four characteristic properties: (i) the optimization problem is often ill-posed due to parameter non-identifiability; (ii) optimization is computationally intensive, involving tens to hundreds of estimated parameters, yet the problems do not qualify as “high-dimensional” problems in the broader optimization literature, since, for example, there is rarely concern that the Hessian cannot be stored in memory; (iii) computation time for numerically solving the optimization problem is dominated by evaluation of the objective function and its derivatives whereas computation time required for a proposed parameter update itself is negligible; (iv) since models are inexact and experimental data is noisy, the residual values between simulation and data may be much larger than zero, even at the global minimum of the optimization problem (such problems are commonly called non-zero residual problems). The existing benchmarks for general purpose optimization, such as the CUTE(r/st) [11–13] set of benchmarks, do not cover models having these four characteristics. Thus, domain-specific benchmarks are required to select optimal optimization algorithms.

Only two collections of models and accompanying experimental data have been proposed as benchmark problems in the literature to date. Villaverde *et al*. [14] proposed a set of 6 published models covering metabolic, developmental and signaling models in different organisms, but only two problems include real experimental data. More recently, Hass *et al*. [15] proposed a set of 20 published models covering signal transduction, immunological regulation, and epigenetic effects in a variety of organisms, all with real experimental data. The models in Hass corpus are small to medium sized, making them computationally tractable, but they are biologically realistic and the basis of a wide variety of previously published biological discoveries. The data are also realistic in their inclusion of Western blots, flow cytometry, and immunofluorescence microscopy. As mentioned above, such data typically provide indirect measurements of a subset of molecular species. Moreover, measurements are noise-corrupted and limited in time resolution, necessitating the use of data from multiple experimental conditions and the introduction of parameter dependent observable functions. Unfortunately, this prohibits the application of more efficient calibration techniques, such as quasi-linearization methods [16, 17] that require direct observation of all model species. Both the structure of biochemical models and limitations in the data impose a non-identifiability that results in parameter optimization problems that are not *well-posed* in a mathematical sense, violating a crucial assumption of many general-purpose optimization algorithms. For these reasons, the Hass corpus *et al*. [15] represents a unique a powerful resource for the evaluation of optimization methods for biochemical models under realistic conditions of varying data-richness and parameter identifiability.

Trust-region methods initialized from hundreds to thousands of random initial parameter values (often referred to as “multi-start”) have performed well for a broad set of biochemical ODE models [15, 18]. Trust-region methods are versatile methods that do not make any assumption about underlying model and data structure except that the objective function must be sufficiently smooth. They use local (quadratic) approximations of the objective function to propose parameter updates and then iteratively refine the local neighborhood in which the local approximation is expected to adequately recapitulate the shape of the true objective function, i.e. the trust-region [19]. Popular implementations of trust-region methods are available in the MATLAB optimization toolbox and the scipy Python optimization module. However, for many problems encountered in biology, including low dimensional biochemical models with as few as 20 parameters, these optimizers do not consistently converge to parameter values that yield similar values for the objective function [15], strongly suggesting failure to reach a global optimum—or even a local optimum. For example, the benchmark study by Hass *et al*. performed optimization for a model based on work of Fujita *et al*. [20], which describes Epidermal Growth Factor (EGF)-mediated activation of the Protein Kinase B pathway (also known as the PI3K/AKT pathway). Hass *et al*. found that the difference in negative log-likelihood between the best and second best parameter values was >5, exceeding the statistical threshold for model rejection according to AIC and BIC criteria [21]. Model selection is challenging in these cases, because poor optimizer performance could easily lead to the erroneous rejection of a model if the starting point that yields the best optimization run was omitted.

**Fig 1:**
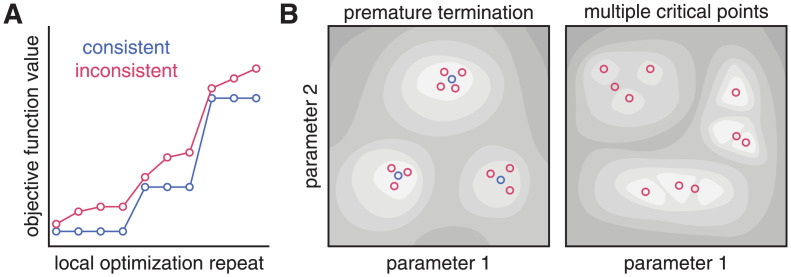
Illustration of final objective function values consistency and possible objective function landscapes. **A**: Waterfall plot with examples of consistent (blue) and inconsistent (red) final objective function values. **B**: Possible objective function landscapes that could explain the waterfall plots in A.

More generally, “optimal” solutions of parameter values that yield inconsistent objective function values, i.e., values that do not cluster in a small set of distinct values (Fig 1A), can indicate either (i) that the optimization converged on a few of the many critical points (local minima, saddle points) (Fig 1B right) or (ii) that the optimization terminated before convergence to any (local) minimum was achieved (Fig 1B left) [22]. Many of the problems of interest in biochemistry and cell biology involve ODE models and datasets that have multiple local minima, which can be a result of curvature of the model manifold [23]. In these cases, repeated convergence of multiple optimization runs on a small set of similar objective function values may not represent a problem with the optimization approach itself, but rather arise from model non-identifiability. This setting contrasts with the situation where the objective function values are inconsistent, despite a large number of runs that converge on one or a set of minima (setting rigorous thresholds for what is considered “consistent” is a tricky problem in and of itself, which we revisit this later in the manuscript). In such a situation, it is unclear whether optimization is non-convergent or the objective function is very “rugged” [3] with many local minima, not all of which may have been identified.

Non-convergent optimization can also result from noisy model simulations, in which lax integration tolerances result in inaccurate numerical evaluation of the value of the objective function and its gradient [24]. Inaccurate gradients often result in poor parameter update proposals, slowing the search in parameter space. Inaccurate objective function values can also result in incorrect rejection of parameter updates, erroneously suggesting convergence to a minimum and leading to premature termination of optimization. For example, Tönsing *et al*. [24] found that optimization runs that yielded similar, but inconsistent objective function values were often located in the neighborhood of the same local minima, suggesting that these runs had been prematurely terminated. The authors suggested that a nudged elastic band method, which aims to identify shortest connecting paths between optima, might be effective in improving consistency.

Premature termination can also arise from problems with the optimization method itself. Dauphin *et al*. [25] found that saddle points are prevalent in the objective functions of neural network models and optimization methods that do not account for directions of negative curvature may perform poorly in the vicinity of saddle points. However, neither the prevalence of saddle points nor their impact on premature optimizer termination has been investigated in the case of biochemical ODE models. Lastly, Transtrum *et al*. [23] suggested that the use of Gauss-Newton Hessian approximations might not work well for sloppy biochemical models. Sloppiness is encountered when the objective function Hessian has a broad eigenvalue spectrum, which results in parameter non-identifiability and an ill-posed optimization problem. Sloppiness is believed to be a universal property of biochemical models [4]. However, the geodesic acceleration proposed by Transtrum *et al*. [23] to address the limitations of Gauss-Newton Hessian approximation has not been widely adopted, likely due to the complexity of its implementation and the computational cost of determining directional second-order derivatives.

Overall, the results described above show that early optimizer termination is a recurrent issue with ODE-based biochemical models and that it has a variety of causes. However, a comprehensive evaluation of this issue on a set of relevant benchmark problems, as well as development and testing of methods to identify or resolve the underlying causes of optimization failures are missing. In principle, this could be addressed by adapting and then combining several optimization algorithms. For example, it might be possible to resolve issues with the Gauss-Newton Hessian approximation by using alternative approximation schemes, such as the Broyden-Fletcher-Goldfarb-Shanno (BFGS) [26–29] scheme. Issues with saddle points could be resolved by employing symmetric rank-one (SR1) [30] approximations that account for negative curvature directions. However, many optimization algorithms were written decades ago and are difficult for practitioners familiar with contemporary programming languages such as Python to customize or extend. Many existing methods lack reporting functions that provide user with statistics about individual optimization traces. These limitations make it difficult to diagnose problems with optimization and to resolve them with algorithmic improvements.

To tackle these and other challenges associated with ODE model optimization, this paper re-implements a standard trust-region algorithm in Python and uses it to study a range of hypotheses about the causes and potential solutions for poor optimizer performance. We find that the use of an inaccurate Hessian approximation is a major contributor to poor optimization performance and therefore propose a novel hybrid Hessian approximation scheme. We demonstrate that this scheme outperforms existing approaches on a the best available corpus of benchmark biochemical network problems.

## 2 Materials and methods

For the purpose of this study, we considered four different optimizers that all implement the interior-trust-region algorithm proposed by Coleman and Li [31]: fmincon, referring to the MATLAB function of the same name and with trust-region-reflective as algorithm and ldl-factorize as subproblem algorithm, lsqnonlin, referring to the MATLAB function of the same name, ls_trf, referring to the scipy function least_squares with trf algorithm, and fides, the new implementation developed in the current manuscript. Below we describe algorithmic and implementation details of fides (a summary is provided in Table 1), and of the benchmark problems we used to evaluate these algorithms.

**Table 1:** Feature overview for different trust-region optimization implementations. The non least-squares column indicates whether the method is applicable to non least-squares problems. The free column indicates whether the implementation is freely available or proprietary software.

Optimizer	Subspace	Non least-squares	BFGS/SR1	Programming Language	free
**lsqnonlin**	$S_{2D}$	□	□	MATLAB	□
**fmincon**	$S_{2D}$	✓	□	MATLAB	□
**ls_trf**	$R^{n_{\theta }}$ , $S_{2D}$	□	□	Python	✓
**fides**	$R^{n_{\theta }}$ , $S_{2D}$	✓	✓	Python	✓

### 2.1 Model formulation

When applied to a biochemical system, an ODE model describes the temporal evolution of abundances of *n*_*x*_ different molecular species *x*_*i*_. The temporal evolution of **x** is determined by the vector field *f* and the initial condition **x**_0_:

$$\begin{array}{c} \dot{x}=f\left(t,x,\theta \right),\;x\left(t_{0}\right)=x_{0}\left(\theta \right). \end{array}$$ (1)

Both *f* and **x**_0_ depend on the unknown parameters $\theta \in \Theta \subset R^{n_{\theta }}$ such as catalytic rates or binding affinities. Restricting optimization to the parameter domain Θ can constrain the parameter search space to values that are realistic based on physicochemical theory and helps prevent numerical integration failures associated with extreme parameter values. For most problems, Θ is the tensor product of scalar search intervals (*l*_*i*_, *u*_*i*_) with lower and upper bounds *l*_*i*_ < *u*_*i*_ that satisfy $l_{i},u_{i}\in R\cup \left\{-\infty ,\infty \right\}$ for every parameter *θ*_*i*_.

Experiments usually provide information about observables **y** which depend on abundances **x** and parameters ***θ***. A direct measurement of **x** is usually not possible. Thus, the dependence of observables on abundances and parameters is described by

$$\begin{array}{c} y(t,\theta )=h(x(t,\theta ),\theta ). \end{array}$$ (2)

**Implementation in this study**: All methods described here use CVODES from the SUNDIALS suite [32] for numerical integration of model equations. CVODES is a multi-step implicit solver for stiff- and non-stiff ODE initial value problems.

### 2.2 Optimization problem

To generate models useful in the study of actual biological systems, model parameters ***θ*** must be inferred from experimental data, which are typically incomplete and subject to measurement noise. A common assumption is that the measurement noise for *n*_*t*_ time-points *t*_*j*_ and *n*_*y*_ observables *y*_*i*_ is additive, independent and normally distributed for all time-points:

$$\begin{array}{c} {\bar{y}}_{ij}=y_{i}\left(t_{j},\theta \right)+\epsilon_{ij},\;\epsilon_{ij}\overset{id}{∼}N\left(0,\sigma_{ij}^{2}\left(\theta \right)\right). \end{array}$$ (3)

Thus, model parameters can be inferred from experimental data by maximizing the likelihood, yielding a maximum likelihood estimate (MLE). However, the evaluation of the likelihood function involves the computation of several products of large terms, which can be numerically unstable. Thus, the negative log-likelihood

$$\begin{array}{c} \begin{array}{cc} J(\theta )= & \frac{1}{2}\sum_{i=1}^{n_{y}}\sum_{j=1}^{n_{t}}\text{log}(2\pi \sigma_{ij}^{2}\left(\theta \right))+{\left(\frac{{\bar{y}}_{ij}-y_{i}\left(t_{j},\theta \right)}{\sigma_{ij}\left(\theta \right)}\right)}^{2} \end{array} \end{array}$$ (4)

is typically used as objective function that is minimized. As the logarithm is a strictly monotonically increasing function, the minimization of *J*(***θ***) is equivalent to the maximization of the likelihood. Therefore, the corresponding minimization problem

$$\begin{array}{c} \theta^{*}=\text{arg}\underset{\theta \in \Theta }{\text{min}}J\left(\theta \right), \end{array}$$ (5)

will infer the MLE parameters. If the noise variance $\sigma_{ij}^{2}$ does not depend on the parameters ***θ***, the objective function (4) has a weighted least-squares formulation. As we discuss later, properties of a least-squares formulation can be exploited in specialized optimization methods. Optimizers that do not require least-squares structure can also work with other noise models [33].

**Implementation in this study**: For the MATLAB optimizers fmincon and lsqnonlin, the objective function and its derivatives were evaluated using data2dynamics [34] (commit b1e6acd), which was also used in the study by Hass *et al*. [15]. For the Python optimizers ls_trf and fides, the objective function and its derivates were evaluated using AMICI [35] (version 0.11.23) and pyPESTO (version 0.2.10).

### 2.3 Trust-region optimization

Trust-region methods minimize the objective function *J* by iteratively updating parameter values ***θ***_*k*+1_ = ***θ***_*k*_ + Δ***θ***_*k*_ according to the local minimum

$$\begin{array}{c} \Delta \theta_{k}=p_{k}^{*}=\underset{p}{\text{argmin}\;}m_{k}\left(p\right)\;s.t.\;∥p∥\leq \Delta_{k} \end{array}$$ (6)

of an approximation *m*_*k*_ to the objective function. Δ_*k*_ is the trust-region radius that restricts the norm of parameter updates. The optimization problem (6) is known as the trust-region subproblem. In most applications, a local, quadratic approximation

$$\begin{array}{c} m_{k}\left(p\right)=f_{k}+g_{k}^{T}p+\frac{1}{2}p^{T}B_{k}p \end{array}$$ (7)

is used, where *f*_*k*_ = *J*(***θ***_*k*_) is the value, *g*_*k*_ = ∇*J*(***θ***_*k*_) is the gradient and *B*_*k*_ = ∇^2^*J*(***θ***_*k*_) is the Hessian of the objective function evaluated at ***θ***_*k*_.

The trust-region radius Δ_*k*_ is updated in every iteration depending on the ratio *ρ*_*k*_ between the predicted decrease $-m_{k}\left(p_{k}^{*}\right)$ and actual decrease in objective function value $\Delta J=J\left(\theta_{k}\right)-J\left(\theta_{k}+p_{k}^{*}\right)$ [19]. The step is accepted if *ρ*_*k*_ exceeds some threshold *μ* ≥ 0. When boundary constraints (on parameter values) are applied, the predicted decrease is augmented by an additional term that accounts for the parameter transformation (see Section 2.6) [31].

**Implementation in this study**: All optimizers evaluated in this study use *μ* = 0 as acceptance threshold. They all increase the trust-region radius Δ_*k*_ by a factor of 2 if the predicted change in objective function value is accurate (*ρ*_*k*_ > 0.75) and the local minimum is at the edge of the trust region ($∥p_{k}^{*}∥>0.9\Delta_{k}$ for fmincon, lsqnonlin and fides, $∥p_{k}^{*}∥>0.95\Delta_{k}$ for ls_trf). All optimizers decrease the trust-region radius if the predicted change in objective function value is inaccurate (*ρ* < 0.25), but fmincon, lsqnonlin and fides set the trust-region radius to $\frac{\text{min}(\Delta_{k},∥p_{k}^{*}∥)}{4}$, while ls_trf sets it to $\frac{\Delta_{k}}{4}$.

When the predicted objective function decrease is negative ($-m_{k}\left(p_{k}^{*}\right)<0$, i.e., an increase in value is predicted) fides and ls_trf set *ρ*_*k*_ to 0.0. Positive values for $m_{k}\left(p_{k}^{*}\right)$ may arise from the augmentation accounting for boundary constraints. Setting *ρ*_*k*_ to 0.0 prevents inadvertent increases to Δ_*k*_ or step acceptance when $-m_{k}\left(p_{k}^{*}\right)$ and Δ*J* are both negative. However, in contrast to fides, ls_trf does not automatically reject respective step proposals and only does so if Δ*J* < 0. fmincon and lsqnonlin only reject step proposals with Δ*J* < 0, but do not take the sign of $m_{k}\left(p_{k}^{*}\right)$ into account when updating Δ_*k*_.

When the objective function cannot be evaluated—for ODE models this is typically the result of an integration failure—all optimizers decrease the trust-region radius by setting it to $\frac{\text{min}(\Delta_{k},∥p_{k}^{*}∥)}{20}$ (fmincon and lsqnonlin), $\frac{\text{min}(\Delta_{k},∥p_{k}^{*}∥)}{4}$ (fides) or $\frac{\Delta_{k}}{4}$ (ls_trf). These subtly nuanced differences in the implementation are likely the result of incomplete specification of the algorithm in the original publication [31]. In particular, handling of $m_{k}\left(p_{k}^{*}\right)>0$, which does not occur for standard trust-region methods, was not described and developers needed to independently work out custom solutions.

### 2.4 Hessian approximation

Constructing the local approximation (7) that defines the trust-region subproblem (6) requires the evaluation of the gradient *g*_*k*_ and Hessian *B*_*k*_ of the objective function at the current parameter values ***θ***_*k*_. While the gradient *g*_*k*_ can be efficiently and accurately computed using first order forward or adjoint sensitivity analysis [36], it is computationally more demanding to compute the Hessian *B*_*k*_ [37]. Therefore several approximation schemes have been proposed that approximate *B*_*k*_ using first order sensitivity analysis. In the following we will provide a brief description of approximation schemes considered in this study, an overview of schemes and their characteristics is provided in Table 2.

**Table 2:** Overview of properties of different Hessian approximation schemes. BFGS is the Broyden-Fletcher-Goldfarb-Shannon algorithm. SR1 is the Symmetric Rank-one update. GN is the Gauss-Newton approximation. SSM is the Structured Secant Method. TSSM is the Totally Structured Secant Method. FX is the hybrid method proposed by Fletcher and Xu [45]. GNSBFGS is the Gauss-Newton Structured BFGS method. The *construction column* indicates whether pointwise evaluation is possible or whether iterative construction is necessary. The *positive semi-definite* column indicates whether the approximation preserves positive semi-definiteness given a positive semi-definite initialization.

Scheme	Construction	Positive Semi-Definite	Convergence Requirement	Requires Least-Squares
**BFGS**	iterative	✓	✓	□
**SR1**	iterative	□	✓	□
**GN**	pointwise	✓	∥**r**(θ*)∥ = 0	✓
**SSM**	pointwise + iterative	□	✓	✓
**TSSM**	pointwise + iterative	□	✓	✓
**FX**	pointwise + iterative	✓	✓	✓
**GNSBFGS**	pointwise + iterative	✓	λ_min_(∇^2^*J*(θ*)) > 0	✓

**Gauss-newton approximation**: The Gauss-Newton (GN) approximation $B_{k}^{\left(GN\right)}$ is based on a linearization of residuals *r*_*ij*_

$$\begin{array}{c} r_{ij}\left(\theta \right)=\frac{{\bar{y}}_{ij}-y_{i}\left(t_{j},\theta \right)}{\sigma_{ij}\left(\theta \right)}\;B_{k}^{\left(GN\right)}=\frac{1}{2}\sum_{i=1}^{n_{y}}\sum_{j=1}^{n_{t}}\nabla r_{ij}\left(\theta_{k}\right)\nabla r_{ij}^{T}\left(\theta_{k}\right), \end{array}$$ (8)

which yields a symmetric and positive semi-definite approximation to *B*_*k*_ and does not account for negative curvature. At the maximum likelihood estimate, *B*^(*GN*)^ is equal to the negative empirical Fisher Information Matrix assuming *σ*_*ij*_ does not depend on parameter values ***θ***. For parameters dependent *σ*, the log(*σ*) term in (4) cannot be assumed to be constant, which results in a non least-squares optimization problem. For non least-squares problems, the adequateness and formulation of the GN approximation is not well established. Thus, Raue [38] proposed to transform the problem into a least-squares form by introducing additional error residuals $r_{ij}^{e}$ and adding a corresponding correction to the Gauss-Newton approximation *B*^(*GN*)^ from (8), yielding *B*^(*GNe*)^:

$$\begin{array}{c} \begin{array}{c} r_{ij}^{e}\left(\theta \right)=\sqrt{2\;\text{log}(\sigma_{ij}\left(\theta \right))+C}\;\\ B_{kl}^{\left(GNe\right)}=B_{kl}^{\left(GN\right)}+\frac{\frac{\partial \sigma_{ij}}{\partial \theta_{l}}\frac{\partial \sigma_{ij}}{\partial \theta_{k}}}{\sigma_{ij}{\left(\theta \right)}^{2}\left(2\;\text{log}\left(\sigma_{ij}\left(\theta \right)\right)+C\right)}, \end{array} \end{array}$$ (9)

where *C* is some arbitrary, but sufficiently large constant so that 2 log(*σ*_*ij*_(***θ***)) + *C* > 0. This condition ensures that residuals are real-valued and the approximation *B*^(*GNe*)^ is positive semi-definite, but inherently makes optimization a non-zero residual problem. Adding the constant *C* to residuals adds a constant to the objective function value and, thus, neither influences its gradient and Hessian nor the location of its minima. However, *C* does enter the GNe approximation, with unclear implications. Instead, Stapor *et al*. [37] suggested that one ignores the second order derivative of the log(*σ*) term in (4), which corresponds to the limit lim_*C*→∞_ *B*^(*GNe*)^ = *B*^(*GN*)^.

**Iterative approximations**: In contrast to the GN approximation, Broyden-Fletcher-Goldfarb-Shanno (BFGS) or Symmetric Rank-one (SR1) are iterative approximation schemes, in which the approximation in the next step

$$\begin{array}{c} \begin{array}{c} B_{k+1}=B_{k}+\Delta \left(s,z,B^{k}\right) \end{array} \end{array}$$

is constructed based on the approximation in the current step *B*_*k*_ and some update Δ(*x*, *s*, *B*^*k*^), where **s** = Δ***θ***_*k*_ and **z** = *g*_*k*+1_ − *g*_*k*_.

The BFGS update scheme

$$\begin{array}{c} \begin{array}{c} \Delta^{\left(BFGS\right)}\left(s,z,M\right)=\frac{zz^{T}}{z^{T}s}-\frac{(Ms){\left(Ms\right)}^{T}}{s^{T}Ms} \end{array} \end{array}$$

guarantees a positive semi-definite approximation as long as a curvature condition **z**^*T*^**s** > 0 is satisfied and the initial approximation $B_{0}^{\left(BFGS\right)}$ is positive semi-definite [19]. Thus, the update scheme is usually only applied with line search methods that guarantee satisfaction of the curvature condition by selecting the step length according to (strong) Wolfe conditions [19]. However, BFGS can also be used in trust-region methods by rejecting updates when the curvature condition is not satisfied, although this invalidates some theoretical convergence guarantees [19].

The SR1 update scheme

$$\begin{array}{c} \begin{array}{c} \Delta^{\left(SR1\right)}\left(s,z,M\right)=\frac{(z-Ms){\left(z-Ms\right)}^{T}}{{\left(z-Ms\right)}^{T}s} \end{array} \end{array}$$

can also yield indefinite approximations, incorporating negative curvature information, and has no step requirements beyond ensuring that the denominator of the update is non-zero.

**Structured secant approximations**: The accuracy of the GN approximation depends on the magnitude of the residuals, since the approximation error is the sum of products of the residuals and the residual Hessians

$$\begin{array}{c} \begin{array}{c} B_{k}=B_{k}^{\left(GN\right)}+\frac{1}{2}\sum_{i=1}^{n_{y}}\sum_{j=1}^{n_{t}}r_{ij}\left(\theta_{k}\right)\nabla^{2}r_{ij}\left(\theta_{k}\right). \end{array} \end{array}$$ (10)

For non-zero residual problems, in which there are indices *i*, *j* such that *r*_*ij*_(θ*) ≫ 0, or in the presence of strong non-linearities, the second order term in (10) does not vanish and the GN approach is known to perform poorly; it can even diverge [39, 40]. This issue is addressed in structured secant methods [39, 41, 42], which combine the pointwise GN approximation with an iterative BFGS approximation *A*_*k*_ of the second order term. In the Structured Secant Method (SSM) [42], the matrix *A*_*k*_ is update using a BFGS scheme:

$$\begin{array}{cc} B_{k+1}^{\left(SSM\right)} & =B_{k+1}^{\left(GNe\right)}+A_{k+1}\\ A_{k+1} & =A_{k}+\Delta^{\left(BFGS\right)}(s,z^{\#},B_{k+1}^{\left(GNe\right)}+A_{k})\\ z^{\#} & ={\left(\nabla r\left(\theta_{k+1}\right)-\nabla r\left(\theta_{k}\right)\right)}^{T}r\left(\theta_{k+1}\right). \end{array}$$

Similarly, the Totally Structured Secant Method (TSSM) [43] scales *A*_*k*_ with the residual norm, to mimic the product structure in the second order term in (10), and, accordingly, scales the update to *A*_*k*_ with the inverse of the residual norm:

$$\begin{array}{cc} B_{k+1}^{\left(TSSM\right)} & =B_{k+1}^{\left(GNe\right)}+∥r\left(\theta_{k+1}\right)∥A_{k+1}\\ A_{k+1} & =A_{k}+\frac{\Delta^{\left(BFGS\right)}(s,z^{†},B_{k+1}^{\left(GNe\right)})+∥r\left(\theta_{k+1}\right)∥A_{k})}{∥r(\theta_{k+1})∥}\\ z^{†} & =B_{k+1}^{\left(GNe\right)}s+z^{\#}\frac{∥r(\theta_{k+1})∥}{∥r(\theta_{k})∥}. \end{array}$$

Despite the use of a BFGS updating scheme, the SSM and TSSM approximations do not preserve positive semi-definiteness, as the matrix $B_{k}^{\left(GNe\right)}$ is updated at every iteration without any additional safeguards. Structured secant approximations have been popularized by the NL2SOL toolbox [44], but are not featured in the standard optimization libraries in MATLAB or Python.

**Hybrid schemes**: Other hybrid schemes can dynamically switch between GN approximations and iterative updates when some metric indicates that the considered problem has non-zero residual structure. For example, Fletcher and Xu [45] proposed an approach to detect non-zero residuals by computing the normalized change in the residual norm and applying BFGS updates when the change is smaller than some tolerance *ϵ*_*FX*_:

$$\begin{array}{c} \begin{array}{cc} B_{k+1}^{\left(FX\right)} & =\{\begin{array}{cc} B_{k}^{\left(FX\right)}+\Delta^{\left(BFGS\right)}\left(s,z,B_{k}^{\left(FX\right)}\right) & \text{if}\;\frac{∥r\left(\theta_{k}\right)∥-∥r\left(\theta_{k+1}\right)∥}{∥r(\theta_{k})∥}<\epsilon_{FX}\\ B_{k+1}^{\left(GNe\right)} & \text{otherwise.} \end{array} \end{array} \end{array}$$

As $B_{k}^{\left(GNe\right)}$ is positive semi-definite and the BFGS updates preserve this property, the FX approximations are always positive semi-definite.

To address the issue of possibly indefinite approximations in the SSM and TSSM approaches, Zhou and Chen proposed a Gauss-Newton structured BFGS method (GNSBFGS) [40].

$$\begin{array}{c} \begin{array}{cc} B_{k+1}^{\left(GNSBFGS\right)} & =\{\begin{array}{cc} B_{k+1}^{\left(GNe\right)}+A_{k+1} & \text{if}\;\frac{z_{k+1}^{◊T}s_{k+1}}{s_{k+1}^{T}s_{k+1}}>\epsilon_{GNSBFGS}\\ B_{k+1}^{\left(GNe\right)}+∥r\left(\theta_{k+1}\right)∥I & \text{otherwise} \end{array}\\ A_{k+1} & =\{\begin{array}{cc} A_{k}+\Delta^{\left(BFGS\right)}(s,z^{◊},A_{k}) & \text{if}\;\frac{z_{k+1}^{◊T}s_{k+1}}{s_{k+1}^{T}s_{k+1}}>\epsilon_{GNSBFGS}\\ A_{k} & \text{otherwise} \end{array}\\ z^{◊} & =z^{\#}\frac{∥r(\theta_{k+1})∥}{∥r(\theta_{k})} \end{array} \end{array}$$

that combines the TSSM approach with the dynamic updating of the FX approach. As sum of two positive semi-definite matrices, $B_{k+1}^{\left(GNSBFGS\right)}$ is always positive semi-definite. The authors demonstrate that the term $\frac{z_{k+1}^{◊T}s_{k+1}}{s_{k+1}^{T}s_{k+1}}$ plays a similar role as the $\frac{∥r\left(\theta_{k}\right)∥-∥r\left(\theta_{k+1}\right)∥}{∥r(\theta_{k})∥}$ term in the FX algorithm. However, they only prove convergence if the tolerance 2*ϵ*_*GNSBFGS*_ is smaller than the smallest eigenvalue λ_min_(∇^2^*J*(θ*)) of the Hessian at the optimal parameters and if ∇^2^*J*(θ*) is positive definite. This condition is not met for problems with singular Hessians, which are often observed for non-identifiable problems.

**Implementation in this study**: fmincon and lsqnonlin were only evaluated using the GNe approximation, as implemented in data2dynamics. ls_trf can only be applied using the GNe approximation. Fides was evaluated using BFGS and SR1 using respective native implementations in addition to GN and GNe, as implemented in AMICI. We used the default value of *C* = 50 for the computation of GNe in both data2dynamics and AMICI. We provide implementations for BFGS, SR1, SSM, TSSM, FX and GNSBFGS schemes in fides. FX, SSM, TSSM and GNSBFGS were applied using GNe, as they require a least-squares problem structure. Hyperparameters *ϵ*_*FX*_ = 0.2 and *ϵ*_*GNSBFGS*_ = 10^−6^ were picked based on recommended values in respective original publications.

### 2.5 Solving the trust-region subproblem

In principle, the trust-region subproblem (6) can be solved exactly [19]. Moré proposed an approach using eigenvalue decomposition of *B*_*k*_ [46]. Yet, Byrd *et al*. [47] noted the high computational cost of this approach and suggested an approximate solution by solving the trust-region problem over a two dimensional subspace $S_{2D}$, spanned by gradient *g*_*k*_ and Newton $B_{k}^{-1}g_{k}$ search directions, instead of $R^{n_{\theta }}$. Yet, for objective functions requiring numerical integration of ODE models, the cost of eigenvalue decomposition is generally negligible for problems involving fewer than 10^3^ parameters.

A crucial issue for the two-dimensional subspace approach are problems with indefinite (approximate) Hessians. For an indefinite *B*_*k*_, the Newton search direction may not represent a direction of descent. This can be addressed by dampening *B*_*k*_ [19], but for boundary-constrained problems additional considerations arise and require the identification of a direction of strong negative curvature [31].

**Implementation in this study**: fmincon and lsqnonlin implement optimization only over $S_{2D}$, where the Newton search direction is computed using preconditioned direct factorization. For preconditioning and direct factorization, fmincon and lsqnonlin employ Cholesky and QR decomposition respectively, which both implement dampening for numerically singular *B*_*k*_. fides and ls_trf implement optimization over $S_{2D}$ (denoted by 2D in text and figures) and $R^{n_{\theta }}$ (denoted by ND in text and figures). For ls_trf, we specified tr_solver = “lsmr” for optimization over $S_{2D}$. To compute the Newton search direction, ls_trf and fides both use least-squares solvers, which is equivalent to using the Moore-Penrose pseudoinverse. fides uses the direct solver scipy.linalg.lstsq, with gelsd as LAPACK driver, while ls_trf uses the iterative, regularized scipy.sparse.linalg.lsmr solver. In fides, the negative curvature direction of indefinite Hessians is computed using the eigenvector to the largest negative eigenvalue (computed using scipy.linalg.eig).

### 2.6 Handling of boundary constraints

The trust-region region subproblem (6) does not account for boundary constraints, which means that ***θ***_*k*_ + Δ***θ***_*k*_ may not satisfy these constraints. For this reason, Coleman and Li [31] introduced a rescaling of the optimization variables depending on how close the current values are to the parameter boundary ∂Θ. This rescaling is realized through a vector

$$\begin{array}{c} \begin{array}{cc} v_{i}\left(\theta \right) & =\{\begin{array}{cccc} \theta_{i}-u_{i} & \text{if}\;\nabla J{\left(\theta \right)}_{i}<0 & \;\text{and}\;u_{i} & <\infty \\ \theta_{i}-l_{i} & \text{if}\;\nabla J{\left(\theta \right)}_{i}\geq 0 & \;\text{and}\;l_{i} & >-\infty \\ -1 & \text{if}\;\nabla J{\left(\theta \right)}_{i}<0 & \;\text{and}\;u_{i} & =\infty \\ 1 & \text{if}\;\nabla J{\left(\theta \right)}_{i}\geq 0 & \;\text{and}\;l_{i} & =-\infty \end{array} \end{array} \end{array}$$

which yields transformed optimization variables

$$\begin{array}{c} \hat{\theta_{k}}=D_{k}^{-1}\theta_{k}=\text{diag}\left(|v\left(\theta_{k}\right)\right){{|}^{\frac{1}{2}})}^{-1}\theta_{k} \end{array}$$

and a transformed Hessian

$$\begin{array}{c} \hat{B_{k}}=D_{k}B_{k}D_{k}+\text{diag}\left(g_{k}\right)\nabla v\left(\theta_{k}\right). \end{array}$$ (11)

As the second term in (11) is positive semi-definite, this handling of boundary constraints can also regularize the trust-region sub-problem, although this is not its primary intent.

Coleman and Li [31] also propose a stepback strategy in which solutions to (6) are reflected at the parameter boundary ∂Θ. Since this reflection defines a one-dimensional search space, the local minimum can be computed analytically at negligible computational cost. To ensure convergence, Δ***θ***_*k*_ is then selected based on the lowest *m*_*k*_(*p*) value among (i) the reflection of p* at the parameter boundary (ii) the constrained Cauchy step, which is the minimizer of (7) along the gradient that is truncated at the paramtere boundary and (iii) p* truncated at the parameter boundary.

**Implementation in this study**: fmincon, lsqnonlin and ls_trf all implement rescaling, but only allow for a single reflection at the boundary [48]. In contrast, fides implements rescaling and also allows for a single or arbitrarily many reflections until the first local minimum along the reflected path is encountered.

### 2.7 Optimizer performance evaluation

To evaluate optimization performance, Hass *et al*. [15] computed the success count *γ*, which represents the number of “successful” optimization runs that reached a final objective function value sufficiently close (difference smaller than some threshold *τ*) to the lowest objective function value found by all methods, and divided that by the time to complete all optimization runs *t*_*total*_, a performance metric that was originally introduced by Villaverde *et al*. [49]. In this study, we replaced *t*_*total*_ with the total number of gradient evaluations across all optimization runs *n*_*grad*_ for any specific optimization setting. The resulting performance metric *ϕ* = *γ*/*n*_*grad*_ ignores differences in computation time for gradients having different parameter values and prevents computer or simulator performance, node load and parallelization from influencing results. This provides a fairer evaluation of the algorithm or method itself and is particularly relevant when optimization is performed on computing clusters with heterogeneous nodes or when different number of threads are used to parallelize objective function evaluation, as it was the case in this study. Since *ϕ* ignores potentially higher computation times for step-size computation, we confirmed that step-size computation times were negligible as compared to numerical integration of model and sensitivity equations. For all trust-region optimizers we studied, the number of gradient evaluations was equal to the number of iterations, with the exception of ls_trf which only uses objective function evaluations when a proposed step is rejected. Thus, 1/*n*_*grad*_ is equal to the average number of iterations for the optimization to converge (divided by the number of optimization runs, which is 10^3^ in all settings). We therefore refer to *ν* = 1/*n*_*grad*_ as the convergence rate. Performance *ϕ* is equal to the product of *γ* and *ν*.

To calculate *γ*, we used a threshold of *τ* = 2, which corresponds to the upper limit of the objective function value in cases in which a model cannot be rejected according to the AIC [50]. Similar to Hass *et al*. [15], we found that changing this convergence threshold did not have a significant impact on performance comparison, but provide analysis for values *τ* = 0.05 (threshold used by Hass *et al*., divided by two to account for difference in objective function scaling, Fig A in S1 Text) and *τ* = 5 (the threshold for rejection according to the AIC and BIC [21], Fig B in S1 Text) in the Supplementary Material.

### 2.8 Extension of boundary constraints

For some performance evaluations, we extended parameter boundaries. Even though initial points are usually uniformly sampled in Θ, we did not modify the locations of initial points when extending bounds. The *Schwen* (Table 3), problem required a different approach in which the bounds for the parameter fragments were not modified, as values outside the standard bounds were implausible.

**Table 3:** Summary of problem characteristics for benchmark examples, as characterized by Hass *et al*. [15].

Problem	*n*_*θ*_	*n*_*x*_	*n*_*y*_ ⋅ *n*_*t*_	sloppy	identifiable
***Bachmann***	113	36	541	✓	□
***Beer***	72	4	27132	✓	□
***Boehm***	9	8	48	✓	✓
***Brannmark***	22	9	43	✓	□
***Bruno***	13	7	77	□	✓
***Crauste***	12	5	21	✓	□
***Fiedler***	19	6	72	✓	□
***Fujita***	19	9	144	✓	□
***Isensee***	46	25	687	✓	□
***Lucarelli***	84	43	1755	✓	□
***Schwen***	30	11	286	✓	□
***Weber***	36	7	135	✓	□
***Zheng***	46	15	60	✓	□

As previously reported [15], extending boundaries can expose additional minima having globally lower objective function values. Thus, success count *γ* for optimization settings with normal boundaries were computed using the lowest objective function *J*_min_ found among all settings excluding those with extended boundaries. *γ* for optimization settings using extended boundaries were computed using the minimum of *J*_min_ and the lowest objective function value found for that particular setting.

### 2.9 Statistical analysis of optimizer traces

During the statistical analysis of optimizer traces, we quantified several numerical values derived from numerical approximation of matrix eigenvalues with limited accuracy (due, for example, to limitations in floating point precision). It was therefore necessary to account for this limitation in numerical accuracy:

**Singular Hessians**: To numerically assess matrix singularity of Hessian approximations, we checked whether the condition number, computed using the numpy function numpy.linalg.cond, was larger than the inverse of the floating point precision *ϵ* = 1/numpy.spacing(1).

**Negative eigenvalues**: To numerically assess whether a matrix has negative eigenvalues we computed the smallest (λ_min_) and largest (λ_max_) eigenvalues of the untransformed Hessian approximation *B*_*k*_ using numpy.linalg.eigvals and checked whether the smallest eigenvalue had a negative value that exceeded numerical noise λ_min_(*B*_*k*_) < −*ϵ* ⋅ |λ_max_(*B*_*k*_)|.

### 2.10 Implementation

fides is implemented as modular, object-oriented Python code. The subproblem, subproblem solvers, stepback strategies and Hessian approximations all have class-based implementations, making it easy to extend the code. Internally, fides uses SciPy [51] and NumPy [52] libraries to store vectors and matrices and perform linear algebra operations. To ensure access to state-of-the-art simulation and sensitivity analysis methods, we implemented an interface to fides in the parameter estimation toolbox pyPESTO, which uses AMICI [35] to perform simulation and sensitivity analysis via CVODES [32]. This approach also enabled import of biological parameter estimation problems specified in the PEtab [53] format.

### 2.11 Benchmark problems

To evaluate the performance of different optimizers, we use the benchmark problems (Table 3) introduced by Hass *et al*. [15]. As discussed in the introduction, these problems are excellent representatives of ODE-based biochemical models and include realistic experimental data for model calibration. We selected a subset of 13 out of the 20 models based on whether they can be encoded in the PEtab [53] format and imported in AMICI and pyPESTO. The exclusion of some models in Hass *et al*. [15] does not reflect a limitation of fides itself, as it supports optimization for any objective function that provides routines to compute its gradient. In summary, the *Hass* problem was excluded because it includes negative initial simulation time, which is not supported by PEtab; the *Raia* model because it involves state-dependent value for *σ*, which is unsupported by AMICI; *Merkle* and *Sobotta* because they are missing SBML implementations; *Swameye* because it includes spline functions that are not supported by SBML; *Becker* because it involves multiple models, which is not supported by pyPESTO; and *Chen* because forward sensitivity analysis is prohibitively computationally expensive for this model. There is no evidence that the excluded models are different in any systematic way from the models we do consider.

All benchmarks problems were previously published and included experimental data for model calibration as described by Table 3, which also provides a brief summary of numerical features of the various benchmarks. These problems cover a wide array of common model features such as preequilibration, log-transformation of observables as well as parameter dependent initial conditions, observable function and noise models. A more detailed description of the biochemical systems described by these models is available in the supplemental material of the study by Hass *et al*. [15].

### 2.12 Simulation and optimization settings

We encountered difficulties reproducing some of the results described by Hass *et al*. [15] and therefore repeated evaluations using the latest version of data2dynamics. We deactivated Bessel correction [15] and increased the function evaluation limit to match the iteration limit. Relative and absolute integration tolerances were set to 10^−8^. The maximum number of iterations for optimization was set to 10^5^. Convergence criteria were limited to step sizes with a tolerance of 10^−6^, and ls_trf code was modified such that the convergence criteria matched the implementation in other optimizers. For all problems, we performed 10^3^ optimizer runs. To initialize optimization, we used the initial parameter values provided by Hass *et al*. [15].

### 2.13 Parallelization and cluster infrastructure

Optimization was performed on the O2 Linux High Performance Compute Cluster at Harvard Medical School, a typical academic High Performance Compute resource running SLURM. O2 includes 390+ compute nodes and 12,000+ compute cores, with the majority of the compute nodes built on Intel architecture; all nodes run CentOS 7.7.1908 Linux with MATLAB R2017a and python 3.7.4. Optimization for each model and each optimizer setting was run as a separate job. For MATLAB optimizers, optimization was performed using a single core per job. For Python optimizers, execution was parallelized on up to 12 cores. Parallelization was always carried out on an individual node, avoiding inter-node communication.

We observed severe load balancing issues due to skewed computational cost across optimization runs for *Bachmann*, *Isensee*, *Lucarelli* and *Beer* models. To mitigate these issues, optimization for these models was parallelized over 3 threads using pyPESTOs MultiThreadEngine and simulation was parallelized over 4 threads using openMP multithreading in AMICI, resulting in a total parallelization over 12 threads. For the remaining models, optimization was parallelized using 10 threads without parallelization of simulations. Wall-time for each job was capped at 30 days (about 1 CPU year), which was only exceeded by the GNSBFGS and FX Hessian approximation schemes for the *Lucarelli* problem after 487 and 458 optimization runs respectively and also by the SSM Hessian approximation scheme for the *Isensee* problem after 973 optimization runs. Subsequent analysis was performed using partial results for those settings.

Compute times could not be reliably compared across methods and problems because it was necessary to use different degrees of parallelization for different problems and optimizations were run on different nodes with distinct processor models. We therefore evaluated performance based on the number of optimizer iterations. The number of iterations is independent of the degree of parallelization and processor models, but for any given implementation, the compute time will be proportional to the number of optimizer iterations.

## 3 Results

### 3.1 Validation and optimizer comparison

The implementation of trust-region optimization involves complex mathematical operations that can result in error-prone implementations. To validate the trust-region methods implemented in fides, we compared the performance of optimization using GN and GNe schemes against implementations of the same algorithm in MATLAB (fmincon, lsqnonlin) and Python (ls_trf). The subspace solvers and Hessian approximation used for each analysis are denoted by the notation *implementation* *subspace*/*hessian*.

**Fig 2:**
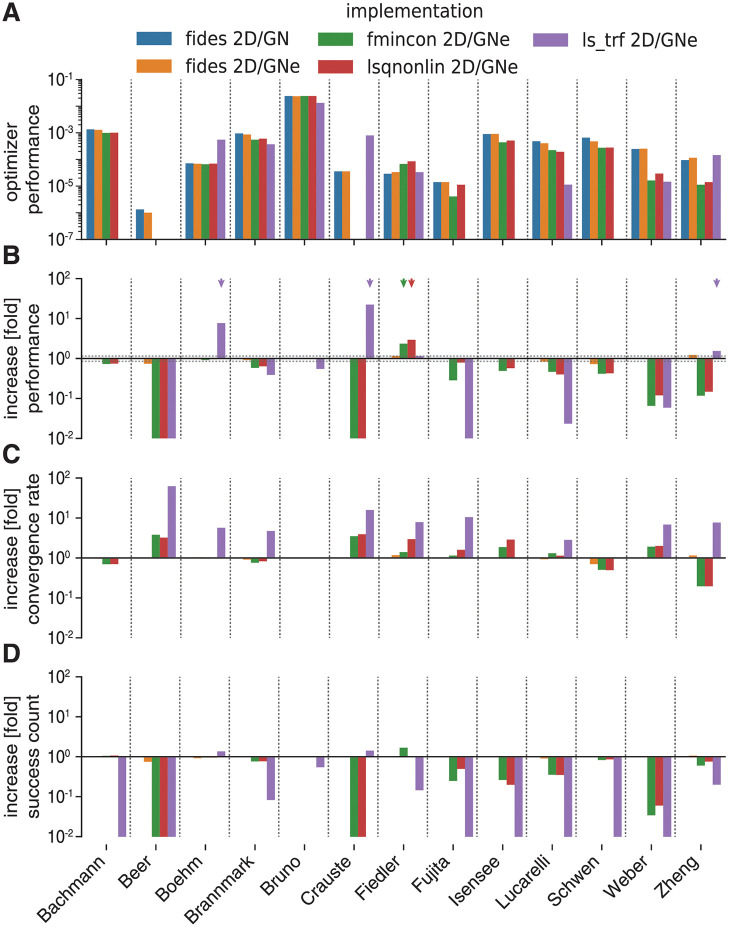
Comparison of MATLAB and Python optimizers. Colors indicate optimizer setting and are the same in all panels. **A**: Performance comparison (*ϕ*), absolute values. **B**: Performance comparison (*ϕ*), values relative to fides 2D/GN. **C**: Increase in convergence count *γ*, values relative to fides 2D/GN. **D**: Increase in convergence rate *ν*, values relative to fides 2D/GN.

We found that fides 2D/GN (blue) and fides 2D/GNe (orange) were the only methods that had non-zero performance (*ϕ* > 0) for all 13 benchmark problems (Fig 2A), with small performance differences between the two methods (0.72 to 1.12 fold difference, average 0.96). This established fides 2D/GN as good reference implementation. In what follows we therefore report the performance of other methods relative to fides 2D/GN (Fig 2B). The ls_trf method outperformed fides 2D/GN on three problems (1.54 to 22.4-fold change; *Boehm*, *Crauste*, *Zheng*; purple arrows), had similar performance on one problem (1.15-fold change; *Fiedler*), exhibited worse performance on four problems (0.02 to 0.55-fold change; *Brannmark*, *Bruno*, *Lucarelli*, *Weber*) and did not result in successful runs (zero performance *ϕ* = 0) for the remaining five problems (*Bachmann*, *Beer*, *Fujita*, *Isensee*, *Schwen*). Decomposing performance improvements *ϕ* into increases in convergence rate *ν* (Fig 2C) and success count *γ* (Fig 2D) revealed that increase in *ϕ* was primarily due to higher *ν*, which was observed for all but four problems (*Bachmann*, *Bruno*, *Isensee*, *Schwen*). However, in most cases, improvements in *ν* were canceled out by larger decreases in *γ*.

For fmincon (green) and lsqnonlin (red), *ϕ* was higher for one problem (2.34 to 2.94 -fold change; *Fiedler*; red/green arrow), similar for two problems (0.92 to 1.00 fold change, *Boehm*, *Bruno*) and worse for the remaining 10 problems (0.00 to 0.80 fold change, *Bachmann*, *Brannmark*, *Fujita*, *Isensee*, *Lucarelli* *Schwen*, *Weber*, *Zheng*), with zero performance on two problems (*Beer*, *Crauste*) (Fig 2B). Since we observed similar *ϕ* for fides 2D/GN (blue) and fides 2D/GNe (orange) on all problems, the differences in *ϕ* between fides 2D/GN and the other implementations are unlikely to reflect use of GNe as opposed to a GN scheme. Instead we surmised that the differences were due to discrepancies in implementation of the Newton direction (this would explain the similarity for the two identifiable problems (*Boehm*, *Bruno*, Table 3)).

Overall, these findings demonstrated that trust-region optimization implemented in fides was more than competitive with the MATLAB optimizers fmincon and lsqnonlin and the Python optimizer ls_trf, outperforming them on a majority of problems. Simultaneously, our results demonstrate a surprisingly high variability in optimizer performance among methods that implement fundamentally similar mathematical operations (i.e., the same algorithm). This variability may explain some of the conflicting findings in previous studies that assumed that differences in optimizer performance arose from the “mathematics” rather than the computational implementation [54, 55].

### 3.2 Parameter boundaries and stepback strategies

**Fig 3:**
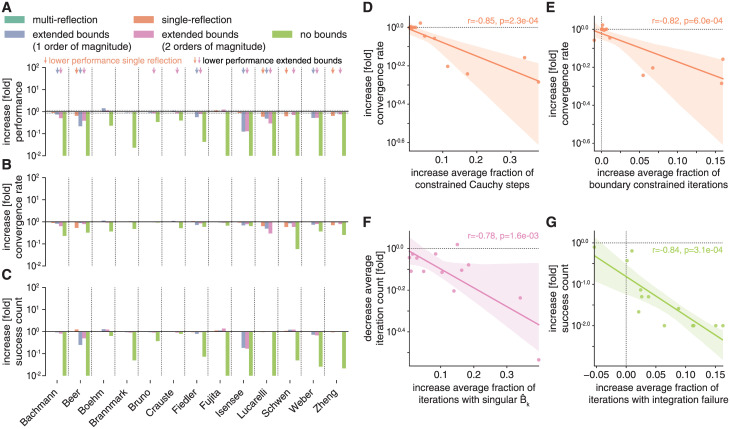
Evaluation of stepback strategies. Colors indicate optimizer setting and are the same in all panels. All increases/decreases are relative to multi-reflection fides 2D/GN with normal bounds. **A**: Performance comparison (*ϕ*). **B**: Increase in convergence count *γ*. **C**: Increase in convergence rate *ν*. **D**: Association between increase in average fraction of constrained Cauchy steps with respect to total number of boundary constrained iterations and increase in *ν* for the single-reflection method. **E**: Association between increase in average fraction of boundary constrained iterations and increase in *ν* for the single-reflection method. **F**: Association between increase in average fraction of iterations with a numerically singular transformed Hessian ${\hat{B}}_{k}$ and decrease in *γ* for the multi-reflection method for fides 2D/GN with bounds extended by two orders of magnitude. **G**: Association between increase in average fraction of iterations with integration failures and increase in *γ* for multi-reflection fides 2D/GN without bounds.

One of the few changes in implementation that we deliberately introduced into the fides code was to allow multiple reflections during stepback from parameter boundary conditions [31]. In contrast, ls_trf, lsqnonlin and fmincon only allow a single reflection [48]. The modular design and advanced logging capabilities of fides make it straightforward to evaluate the impact of such modifications on optimizer performance *ϕ* and arrive at possible explanations for observed differences. For example, when we evaluated fides 2D/GN with single (orange) and multi-reflection (dark-green, Fig 3A–3C) implementations and correlated changes to *ϕ* with statistics of optimization trajectories (Fig 3D and 3E), we found that the single reflection performance *ϕ* was reduced on four problems (0.59 to 0.65-fold change; *Beer*, *Lucarelli*, *Schwen*, *Zheng*; orange arrows Fig 3A). Lower performance was primarily due to a decrease in convergence rate *ν* (Fig 3B and 3C). We attributed this behavior to the fact that a restriction on the number of reflections lowered the predicted decrease in objective function values for reflected steps. This, in turn, increased the fraction of iterations in which stepback yielded constrained Cauchy steps (Pearson’s correlation coefficient *r* = −0.85, p-value *p* = 2.3 ⋅ 10^−4^, Fig 3D) as well as the average fraction of boundary-constrained iterations (*r* = −0.82, p = 6 ⋅ 10^−4^, Fig 3E), both slowing convergence.

A naive approach to addressing issues with parameter boundaries is to extend or remove them. We therefore repeated optimization with fides 2D/GN (multi-reflection) with parameter boundaries extended by one (blue) or two (pink) orders of magnitude or completely removed (light green). We found that extending boundaries by one order of magnitude reduced *ϕ* for 6 problems (0.12 to 0.74 fold change; *Bachmann*, *Beer*, *Fiedler*, *Isensee*, *Lucarelli*, *Weber*; blue arrows Fig 3A) and extending boundaries by two orders of magnitude reduced *ϕ* for an additional 4 problems (0.13 to 0.85 fold change; *Bruno*, *Crauste*, *Schwen*, *Zheng*; pink arrows Fig 3A). We found that decreased *ϕ* was primarily the result of lower *ν* (Fig 3B), which we attributed to a larger fraction of iterations in which the transformed Hessian ${\hat{B}}_{k}$ was singular (*r* = −0.78, *p* = 1.6 ⋅ 10^−3^, Fig 3F). Removing boundaries decreased *ϕ* for all problems, a result of lower values of *γ*, which we attributed to higher fraction of iterations with integration failures (*r* = −0.82, *p* = 5.7 ⋅ 10^−4^, Fig 3G).

These findings demonstrate the importance and difficulty of choosing appropriate optimization boundaries, since excessively wide boundaries may lead to frequent integration failures and/or the creation of an ill-conditioned trust-region subproblem. In contrast, using boundaries that are too narrow has the potential to exclude the global optimum. When managing boundary constraints the use of multi-reflection as compared to single-reflection yields a small performance increase, albeit significantly smaller than the variation we observed (in the previous section) between different implementations of the same optimization algorithm.

### 3.3 Iterative schemes and negative curvature

To further study the positive effect of improving the conditioning of trust-region subproblems on the optimizer performance *ϕ*, we carried out optimization using BFGS and SR1 Hessian approximations. BFGS and SR1 can yield full-rank Hessian approximations, resulting in well-conditioned trust-region subproblems, even for non-identifiable problems. Moreover, in contrast to GN, these approximations converge to the true Hessian under mild assumptions [19]. The SR1 approximations can also account for directions of negative curvature and might therefore be expected to perform better when saddle-points are present.

**Fig 4:**
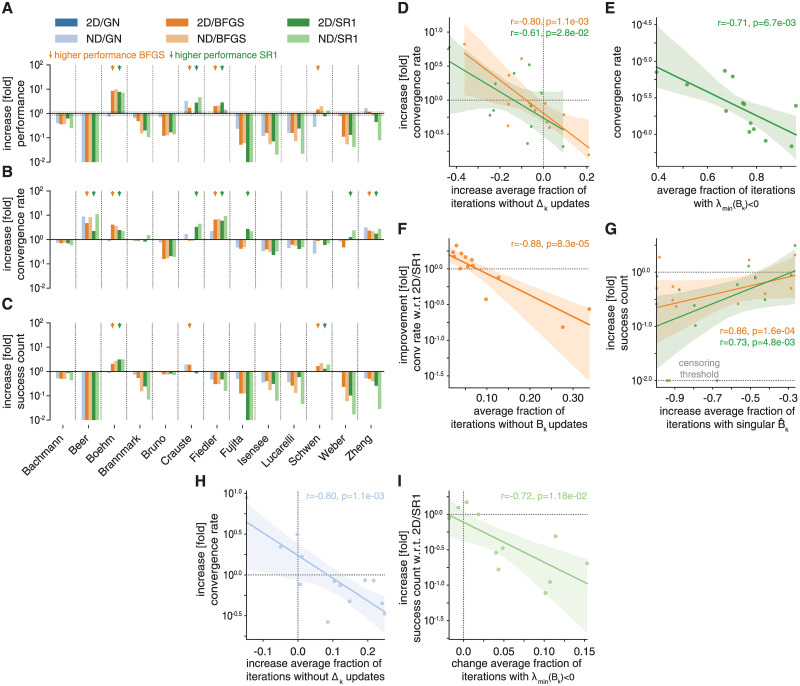
Evaluation of iterative Hessian approximation schemes. Color scheme is the same in all panels. All increases/decreases are relative to fides 2D/GN unless otherwise noted. **A**: Performance comparison (*ϕ*). **B**: Increase in convergence count *γ*. **C**: Increase in convergence rate *ν*. **D**: Association between increase in average fraction of iterations without updates to the trust-region radius Δ_*k*_ and increase in *ν* for fides 2D/SR1 (green) and fides 2D/BFGS (orange). **E**: Association between average fraction of iterations where smallest eigenvalue λ_min_ of the transformed Hessian ${\hat{B}}_{k}^{\left(SR1\right)}$ is negative and *ν* for fides 2D/SR1. **F**: Association between fraction of iterations without updates to *B*_*k*_ and increase in *ν* relative to fides 2D/SR1 for fides 2D/BFGS. **G**: Association between fraction of iterations with a numerically singular transformed Hessian ${\hat{B}}_{k}$ and increase in *γ* for fides 2D/SR1 (green) and fides 2D/BFGS (orange). Change in *γ* was was censored at a threshold of 10^−2^ to visualize models with *γ*. **H**: Association between fraction of iterations without updates to Δ_*k*_ and increase in *ν* for fides ND/GN. **I**: Association between change in average fraction of iterations where smallest eigenvalue λ_min_ of the transformed Hessian ${\hat{B}}_{k}^{\left(SR1\right)}$ is negative and *γ* relative to fides 2D/SR1 for fides ND/SR1.

We compared *ϕ* for fides 2D/GN (blue), fides 2D/BFGS (orange) and fides 2D/SR1 (green) (Fig 4A) and found that fides 2D/BFGS failed to reach the best objective function value for one problem (*Beer*) and Fides 2D/SR1 for two problems (*Beer*, *Fujita*). Compared to fides 2D/GN, *ϕ* for fides 2D/BFGS was higher on three problems (2.02 to 9.88 fold change; *Boehm*, *Fiedler*, *Schwen*; orange arrows) and four problems for fides 2D/SR1 (1.32 to 7.12 fold change; *Boehm*, *Crauste*, *Fiedler*; green arrows), it was lower for a majority of the remaining problems (BFGS 7 of 13 problems, 0.05 to 0.49 fold change; SR1 8 of 13 problems, 0.07 to 0.78 fold change). Decomposing *ϕ* into improvements in convergence rate *ν* (Fig 4B) and improvements in success counts *γ* (Fig 4C) revealed that SR1 improved *ν* for 7 problems (2.23 to 5.97 fold change; *Beer*, *Boehm*, *Crauste*, *Fiedler*, *Fujita*, *Weber*, *Zheng*; green arrows Fig 4B). Out of these 7 problems, BFGS improved *ν* for only four problems (2.33 to 6.63 fold change; *Beer*, *Boehm*, *Fiedler*, *Zheng*; orange arrows Fig 4B). We found that for both approximations, the increase in *ν* was correlated with the change in average fraction of iterations without trust-region radius (Δ_*k*_) updates (BFGS: *r* = −0.8, *p* = 1.1 ⋅ 10^−3^; SR1: *r* = −0.61, *p* = 2.8 ⋅ 10^−2^, Fig 4D). Δ_*k*_ is not updated when the predicted objective function decrease is in moderate agreement with the actual objective function decrease (0.25 < *ρ*_*k*_ < 0.75, see Section 2.3), likely a result of inaccurate approximations to the Hessian. Thus, higher convergence rate *ν* of SR1 and BFGS schemes was likely due to more precise approximation of the objective function Hessian.

To better understand the origins of performance differences between BFGS and SR1, we analysed the eigenvalue spectra of SR1 approximations and found that SR1 convergence rate *ν* was correlated with the average fraction of iterations where the transformed Hessian approximations ${\hat{B}}_{k}$ had negative eigenvalues (*r* = −0.71, *p* = 6.7 ⋅ 10^−3^, Fig 4E). This correlation suggests that directions of negative curvature approximated by the SR1 scheme tended not to yield good search directions; handling of negative curvature is therefore unlikely to explain observed improvements in convergence rates. In contrast to negative eigenvalues, we found that difference in *ν* between BFGS and SR1 was correlated with the average fraction of iterations in which the BFGS approximation did not produce an update (*r* = −0.88, *p* = 8.3 ⋅ 10^−5^, Fig 4F). The BFGS approximation is not updated when the curvature condition is violated (see Section 2.4). Such a high fraction of iterations not prompting updates is surprising, since the violation of the curvature condition is generally considered to be rare [19]. It is nonetheless a plausible explanation for lower convergence rates, since the BFGS approximation is not expected to always converge to the true Hessian under such conditions [19].

For three problems, the BFGS (orange arrows, Fig 4C) and/or SR1 (green arrows, Fig 4C) approximations increased *γ* (1.29 to 3.12 fold change; *Boehm*, *Crauste*, *Schwen*), but for most problems *γ* was reduced by more than two-fold (−∞ to 0.46 fold change; SR1+BFGS: *Beer*, *Fiedler* *Fujita*, *Isensee*, *Lucarelli* *Weber*, *Zheng*; BFGS: *Bachmann*; SR1: *Brannmark*), canceling the benefit of faster convergence rate for many of these problems. Paradoxically, we found that convergence count changes were correlated with changes in the average fraction of iterations having ill-conditioned trust-region subproblems (BFGS: *r* = 0.86, *p* = 1.6 ⋅ 10^−4^; SR1: *r* = 0.73, *p* = 4.8 ⋅ 10^−3^, Fig 4G). Therefore, improved conditioning of the trust-region subproblem, unexpectedly, came at the cost of smaller regions of attraction for minima having low objective function values.

We complemented the analysis of 2D methods by evaluating their respective ND methods, which almost exclusively performed worse than 2D methods. We found that Fides ND/GN outperformed Fides 2D/GN on two problems (1.43 to 3.25-fold change; *Crauste*, *Zheng*) and performed similarly on one problem (0.99-fold change; *Fiedler*). Fides ND/BFGS outperformed Fides 2D/BFGS on three problems (1.16 to 1.37-fold change; *Boehm*, *Fujita*, *Schwen*) and performed similarly on three examples (1.01 to 1.06-fold change; *Bachmann*, *Bruno*, *Fiedler*). Fides ND/SR1 outperformed Fides 2D/SR1 on two problems (1.51 to 1.70-fold change; *Crauste*, *Schwen*). These results were surprising, since the use of 2D methods is generally motivated by lower computational costs, not better performance; the ND approach gives, in contrast to the 2D approach, an exact solution to the trust-region subproblem. For GN, the change in convergence rate *ν* was correlated with the change in average fraction of iterations in which the trust-region radius Δ_*k*_ was not updated (*r* = −0.8, *p* = 1.1 ⋅ 10^−3^, Fig 4H). For fides SR1/ND, the change in *γ* with respect to fides SR1/2D was correlated with the change in average fraction of iterations in which ${\hat{B}}_{k}$ had negative eigenvalues (*r* = −0.72, *p* = 1.2 ⋅ 10^−2^, Fig 4I). This suggests that inaccuracies in Hessian approximations may have stronger impact on ND methods as compared to 2D methods, thereby mitigating advantages that are theoretically possible.

Overall these results suggest that BFGS and SR1 approximations can improve optimization performance through faster convergence, but often suffer from poorer global convergence properties. Thus, they rarely outperform the GN approximation. BFGS and SR1 perform similarly on most problems, with the exception of a few problems for which BFGS cannot be updated due to violation of curvature conditions. We conclude that, while saddle points may be present in some problems, they do not seem to pose a major issues that can be resolved using the SR1 approximation.

### 3.4 Hybrid switching approximation scheme

We hypothesized that the high success count *γ* of the GN approximation primarily arose in the initial phase of optimization, which determines the basin of attraction on which optimization will converge. In contrast, we the high convergence rate *ν* of the BFGS approximation seemed more likely to arise from more accurate Hessian approximation in later phases of optimization, when convergence to the true Hessian is achieved. To test this idea, we designed a hybrid switching approximation that initially uses a GN approximation, but simultaneously constructs an BFGS approximation. As soon as the quality of the GN approximation becomes limiting, as determined by a failure to update the trust-region radius for *n*_*hybrid*_ consecutive iterations, the hybrid approximation switches to the BFGS approximation for the remainder of the optimization run.

**Fig 5:**
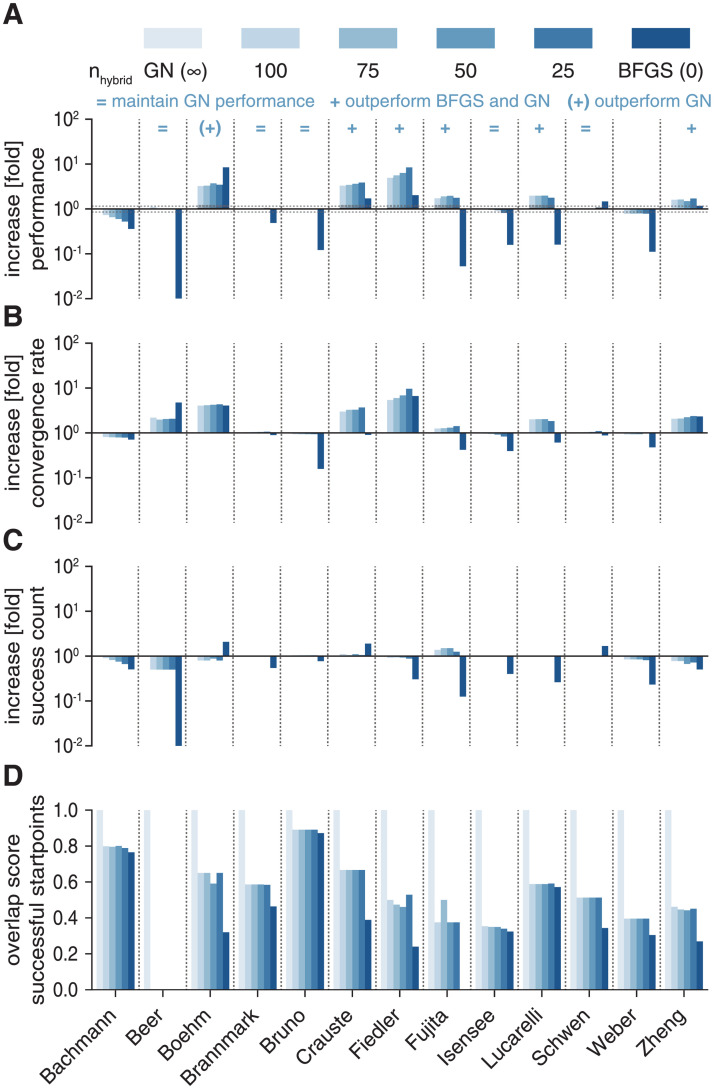
Evaluation of hybrid switching approximation. Color scheme is the same in all panels. All increases/decreases are relative to fides 2D/GN. **A**: Performance comparison (*ϕ*). **B**: Increase in convergence count *γ*. **C**: Increase in convergence rate *ν*. **D**: Overlap score for start-points that yield successful optimization runs with respect to fides 2D/GN.

We compared the hybrid switching approach using different values of *n*_*hybrid*_ (25, 50, 75, 100) to fides 2D/GN (equivalent to *n*_*hybrid*_ = ∞) and fides 2D/BFGS (equivalent to *n*_*hybrid*_ = 0). Evaluating optimizer performance *ϕ*, we found that the hybrid approach was successful for all problems, with *n*_*hybrid*_ = 50 performing best, improving *ϕ* by an average of 1.51 fold across all models (range: 0.56 to 6.34-fold change). The hybrid approach performed better than fides 2D/GN and fides 2D/BFGS on 5 problems (1.71 to 6.34-fold change; *Crauste*, *Fiedler*, *Fujita*, *Lucarelli*, *Zheng*; + signs Fig 5A). It performed better than fides 2D/GN, but worse than fides 2D/BFGS only on one problem (*Boehm*; (+) sign Fig 5A). The hybrid approach performed similar to fides 2D/GN on 5 out of the 7 remaining problems (0.89 to 1.03-fold change; *Beer*, *Brannmark*, *Bruno*, *Isensee*, *Schwen*; = signs Fig 5A). Decomposing *ϕ* into *γ* and *ν*, we found that hybrid switching resulted in higher *ν* for the four problems in which fides 2D/BFGS had higher *ν* than fides 2D/GN (*Beer*, *Boehm*, *Fiedler*, *Zheng*), as well as three additional problems (*Crauste*, *Fujita*, *Lucarelli*). These were the same three problems for which SR1 had higher *ν* (Fig 4B), but BFGS did not, as a consequence of a high number of iterations without *B*_*k*_ updates (Fig 4F). Consistent with this interpretation, we confirmed that the hybrid approach had very few iterations without *B*_*k*_ updates. In contrast to BFGS, the hybrid switching approach maintained a similar *γ* as GN, meaning that higher *ν* generally translated into higher *ϕ*. Evaluating the overlap between start-points that yielded successful runs for GN showed a higher overlap for the hybrid switching approach as compared to BFGS (Fig 5D), suggesting that higher *γ* for the hybrid switching was indeed the result of higher similarity in regions of attraction. These findings further corroborate that local convergence of fides 2D/GN is slowed by the limited approximation quality of GN.

## 4 Comparison of hybrid approximation schemes

Inaccuracies in the GN approximation have been previously discussed in the optimization literature and are known to lead to slow convergence and even divergence of optimization runs [39, 40]. Several methods have been proposed to address this issue in the context of non-zero residual problems. These include the Structured Secant Method (SSM) [42], the Totally Structured Secant Method (TSSM) [43], the hybrid scheme by Fletcher and Xu (FX) [45] and the Gauss-Newton Structured BFGS (GNSBFGS) approach [40]. All of these methods combine the GN and BFGS approximations in different ways (see Section 2.4).

**Fig 6:**
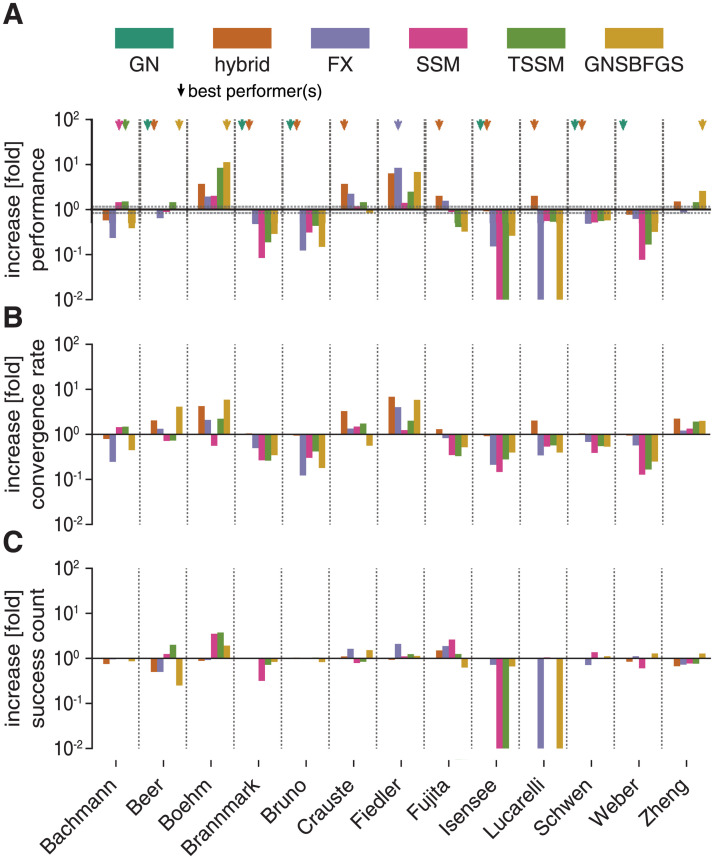
Evaluation of hybrid switching approximation. Color scheme is the same in all panels. All increases/decreases are relative to fides 2D/GN. **A**: Performance comparison (*ϕ*). **B**: Increase in convergence count *γ*. **C**: Increase in convergence rate *ν*.

We implemented support for these Hessian approximation schemes in fides and compared optimizer performance *ϕ* against fides 2D/GN and the best performing hybrid switching method (*n*_*hybrid*_ = 50). We found that the hybrid switching method was among the best performing methods (fold change-0.85 to 1.15) on a majority of problems (7 out of 13; *Beer*, *Brannmark*, *Bruno*, *Crauste*, *Fujita*, *Lucarelli*, *Schwen*; oragne arrows Fig 6A) and was the only method other than fides 2D/GN that resulted in successful runs for all problems. fides 2D/GN was among the best performers on 6 problems (*Beer*, *Boehm*, *Bruno*, *Isensee*, *Schwen*, *Weber*; dark green arrows Fig 6A) whereas GNSBFGS was among the best performers for three problems (*Beer*, *Boehm*, *Zheng*; yellow arrows Fig 6A) and FX for one problem (*Fiedler*; purple arrows Fig 6A). Both GNSBFGS and FX failed for one problem (*Lucarelli*). SSM and TSSM were among the best performers on one problem (*Bachmann*) and failed on one problem (*Isensee*). We conclude that the hybrid switching method is the most reliable and efficient method among all methods that we tested.

## 5 Discussion

In this paper we evaluated the properties of trust-region methods that affect the performance of parameter estimation for ODE models of cellular biochemistry. We used a previously described corpus of 13 models and the associated experimental data as a testbed relevant to many problems encountered in the application of dynamical models in bio-medicine. The evaluation was made possible by the re-implementation of a MATLAB algorithm originally described by Coleman and Li [31]. The resulting fides toolbox also implements advanced logging capabilities that permits detailed analysis of optimization traces. We then compared success counts *γ* and convergence rate *ν* to the numerical properties of optimization traces across multiple models. This analysis promoted us to develop a novel hybrid switching scheme that uses two approaches for Hessian approximation: the Gauss-Newton approximation early in a run (when the basin of attraction is being determined) and the BFGS approximation later in a run (when a fast convergence rate to the local minimum is crucial). For many but not all problems in our test corpus, we found that fides in combination with hybrid switching exhibited the best performance and resolved issues with inconsistent final objective function values.

Overall we were unable to identify a single uniformly superior optimization approach, in line with the infamous “no free lunch” theorem of optimization [10]. Hybrid switching improved average performance and was superior for the majority of problems, but there remained a minority for which other hybrid methods performed substantially better. This heterogeneity highlights the existence of several distinct problem classes but we have thus far been unable to identify their essential properties. We anticipate that future innovation in optimization methods for biochemical models will likely be driven either by better understanding of how differences in optimizer performance relate to model structure, enabling *a priori* selection of the best numerical approach, or new ways of analyzing the numerical properties of optimization traces, driving the development of new adaptive methods. Until then, the availability of multiple Hessian approximation schemes and trust-region sub-problem solvers in fides will be of general utility with a range of models. We have used it ourselves with large ODE-based models that are on the limit of what can be considered practically [56]. Similarly, fides will be a sound foundation for the development of new and better optimization methods.

Our findings suggest that issues previously encountered with fmincon and lsqnonlin are likely due to premature optimizer termination and not due to “rugged” objective function landscapes (a situation in which many similar local minima are present). Our results also corroborate previous findings from others showing that the use of Gauss-Newton approximations can be problematic for optimization problems featuring sloppy models [4]. However, we did not find improved performance with the SR1 scheme, which can handle saddle points. The inconsistent and often poor performance of BFGS and SR1 schemes was also unexpected, but our findings suggest that the problem arises in the global convergence properties of BFGS and SR1, as revealed by lower convergence counts *γ*. Global convergence properties depend on the shape of the objective function landscape and are therefore expected to be problem-specific. BFGS and SR1 may therefore perform better when combined with hybrid global-local methods such as scatter search [57], which substantially benefit from good local convergence [49], but are less dependent on global convergence. Moreover, SR1 and BFGS schemes enable the use of trust-region optimization for problems in which the GN approximation is not applicable, such as when a non-Gaussian error model is used [33] or when gradients are computed using adjoint sensitivities [36], which is particularly relevant for large multi-pathway models with many parameters [58].

We were surprised to observe that differences in the performance of distinct numerical implementations of the same fundamental mathematical instructions (algorithm) could be greater than the differences between distinct algorithms. We propose that these unexpected differences arise from how numerical edge cases are handled. For example, fides uses a Moore-Penrose pseudoinverse to compute the Newton search direction for the 2D subproblem solver, while fmincon uses damped Cholesky decomposition. Another possible source of difference is the use of different simulation and sensitivity computation routines. While both data2dynamics and AMICI employ CVODES [32] for simulation and computation of parameter sensitivity, there may be slight differences in implementation of advanced features such as handling of events and pre-equilibration. Overall, these findings demonstrate the complexity of comparing trust-region methods and the impact of subtle differences in numerical methods on optimizer performance. Thus, the benchmarking of different optimization algorithms requires consistent implementation within a single framework. This consistency is likely to have practical benefit for individuals interested in developing new optimization methods. It is also possible that the superior performance exhibited by fides will generalize to optimization problems other than biochemical models.

Overall, our results demonstrate that fides not only finds better solutions to parameter estimation problems for ODE-based biochemical models when state-of-the-art algorithms fail, but also performs on par or better on problems where established methods find good solutions. The modular and flexible implementation of fides and its interoperability with other toolboxes that facilitates the import of PEtab problems is expected to drive its adoption within the systems biology as a preferred means of performing parameter estimation.

## Supporting information

S1 Text Performance comparison using different values for the consistency thresholds *τ*. (PDF)
