# Supplementary material for: Fides: Reliable trust-region optimization for parameter estimation of ordinary differential equation models
Source: PLoS Comput Biol. 2022 Jul 13;18(7):e1010322. doi: 10.1371/journal.pcbi.1010322 (PMC9312381; doi:10.1371/journal.pcbi.1010322)
Supplement: S1 Text — (PDF) [file pcbi.1010322.s001.pdf]

Supplementary Material to "Fides: Reliable Trust-Region Optimization for Parameter Estimation of Ordinary Differential Equation Models"

Fabian Fröhlich [1],Peter K. Sorger [1]

1 Laboratory of Systems Pharmacology and Department of Systems Biology, Harvard Medical School, Boston, MA 02115, USA

1 Performance comparison using different thresholds τ

We repeated all of the performance comparisons presented in the main manuscript with different inconsistency thresholds τ. For $\tau = 0.05$ we found extremely low convergence counts ($< 1\%$) for several problems, which means that reported performance may be subject to substantial statistical uncertainty. Nevertheless, we included respective results as we believe this choice of threshold is most relevant for tasks such as profile likelihood analysis where even small inconsistencies in convergence can lead to non-smooth profiles or premature termination of the profile computation. As we observed 0 convergence counts for the `fides 2D/GN` method for the *Beer* problem, we used `fides 2D/GNe` as reference in that setting.

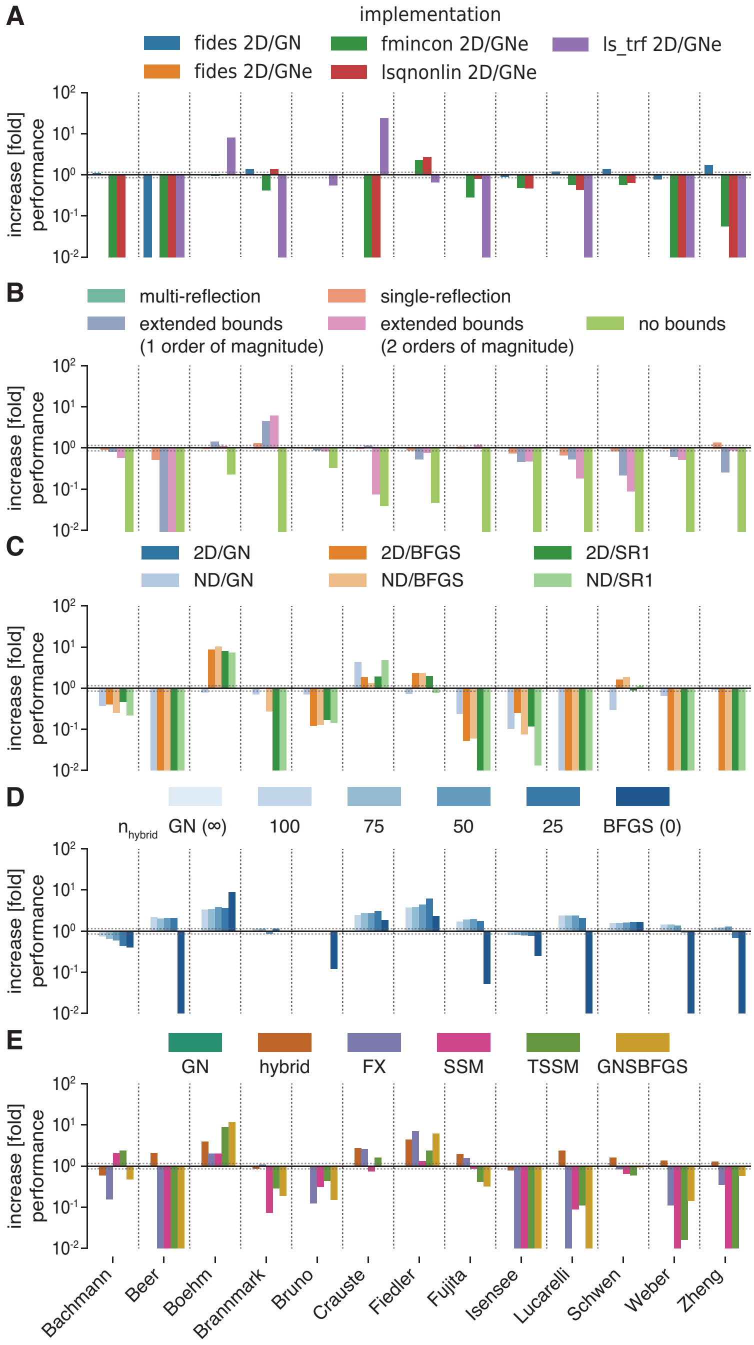

Figure A in Text S1: Performance comparison for the different settings studied in the main manuscript with threshold $\tau = 0.05$. Increase in performance is reported relative to `fides 2D/GNe`. **A:** Comparison with other MATLAB and Python optimizers. **B:** Comparison with different boundary constraint handling strategies. **C:** Comparison with SR1 and BFGS approximation schemes and `ND` subspace solvers. **D:** Comparison of hybrid switching approach. **E:** Comparison of different hybrid approaches.

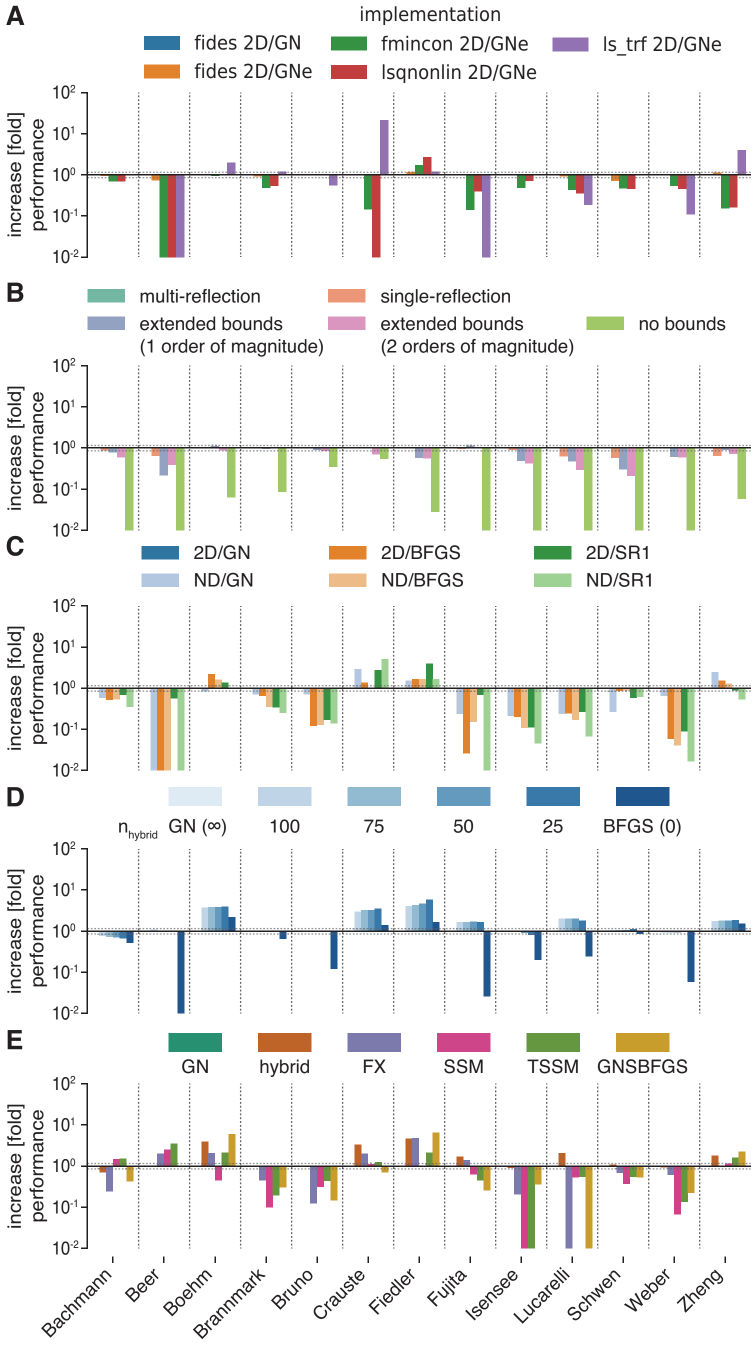

Figure B in Text S1: Performance comparison for the different settings studied in the main manuscript with threshold $\tau = 5$. Increase in performance is reported relative to `fides 2D/GN`. **A:** Comparison with other MATLAB and Python optimizers. **B:** Comparison with different boundary constraint handling strategies. **C:** Comparison with SR1 and BFGS approximation schemes and `ND` subspace solvers. **D:** Comparison of hybrid switching approach. **E:** Comparison of different hybrid approaches.
