# Supplementary material for: Fides: Reliable trust-region optimization for parameter estimation of ordinary differential equation models
Source: PLoS Comput Biol. 2022 Jul 13;18(7):e1010322. doi: 10.1371/journal.pcbi.1010322 (PMC9312381; doi:10.1371/journal.pcbi.1010322)
Supplement: Attachment — Submitted filename: Response to Reviewers.pdf [file pcbi.1010322.s002.pdf]

Response to Reviewers

Unsupervised Deep Learning Supports Reclassification of Bronze Age Cypriot Writing System

Editor's Comments

Reviewer 1 provides a great deal of technical advice worth following, especially concerning a relative lack of clarity and exhaustivity in explaining the methods. One concern that I share with them has to do with the validation of your model on cursive Hiragana, which yields lackluster results, calling into question the subsequent applicability of the model to the Cypro-Minoan data. This, in my view, is the number one issue raised by your paper. Please address it in depth, by explaining why the model may yield reliable conclusions in spite of its limited aplicability to a better known and better documented script, and by qualifying your conclusions accordingly.

Like Reviewer 1, I noticed that your paper was not accompanied by open data and code, and that you declared some restrictions would apply to the sharing of the data. Given the highly technical and innovative nature of your study, I do think that giving Reviewer 1 access to your data and code is important to let them appreciate the robustness of the results.

Reviewer 2 confesses serious misgivings about your raw data, but also notes that your conclusion is plausible, and indeed can be defended on other grounds. Please address their thorough and detailed comments; they are mostly dealing with the quality and completeness of the sources. I share their last remark on the fact the three versions of Cypro-Minoan might be one and the same script, without necessarily encoding one and the same language.

Authors' replies (in bold)

We are grateful and thank the Editor and the Reviewers for their precious insight. It is our contention that this investigation provides independent support to a hypothesis that had already been put forward on the basis of paleography and distributional analysis of signs across the subcorpora of Cypro-Minoan. We hope to have addressed all misgivings of Reviewer 2 both in the new manuscript and in our responses below.

Reviewer #1: The paper is clearly written and has a straightforward research question, which aims at investigating if three subgroups of the Cypro-Minoan script are the same language or not. The methods used in the paper are relevant for the research question and well described. The appreciation toward the paper is generally positive. However, some revisions could be made to clarify more details, which is why the reviewer suggests: Accept with (major?) revisions.

General comments:

- The title mentions 'deep learning' but within the text, the term changes between 'machine learning' (e.g., also the term ‘machine-based techniques on p2) and 'deep learning'. I suggest to synchronize which term to use when referring to the methods. IMHO, both methods are used in the paper, e.g., k-means is more likely to be affiliated to the machine learning category while neural network is more likely to be affiliated to the deep learning category.

We thank the reviewer for this comment and we have made the appropriate changes in the manuscript.

- I understand that the authors have concern to release detailed code and data upon acceptance, but in the current state it is hard to judge how robustly was the analysis conducted. For example, there is not much details about the detailed settings of the parameters and few information from the attached supplementary tables can be used to interpret the robustness of the analysis. The description of the method is well-written though, so the editors may decide if the code is needed for reviewing or not.

We are providing the code (see below) and the data to reproduce our results. Since the size of the data is relatively large, we split it into two archives. In the first one, all the source code is provided and we included pretrained vectors, so that all results obtained by applying the paleographic vector can be reproduced using only this archive. For the models, we provide a separate archive, since they are more than 5GB.

The code is found here:
https://drive.google.com/file/d/1AXL5MSQyw33MjXTthbjN_1swvy5296Bm/view?usp=sharing and in the Readme we provide a link to the models as well.

- P3: Is there a table or a part of text giving the distribution of the three scripts in the used data? I could not find the information in the text or in the supplementary materials (sorry if I missed it). My follow-up question on the distribution would be: If there is a lack of balance in the data, does this lack of balance between the three scripts have an effect on the output of the experiments?

In the revised version of the manuscript, we now provide detailed information about the dataset, including some statistics about the sequences. About the

second part of the question: while there is an imbalance between the three subcorpora, the imbalance is less severe when we only separate the tablets from the rest of the corpus. This imbalance should have little impact on the final results.

- P4-P5: “We tested various supervised and unsupervised deep learning models … Our preliminary experiments found ….” If these additional experiments are mentioned, their procedure and output should probably be provided somewhere, either in the text or in the supplementary materials.

We removed all mentions of these preliminary results, as they are not part of the findings of this paper and they used different datasets which cannot be compared with Sign2Vec$_d$ as it is now.

- P5: “The model therefore tries to reconstruct the category to which the sign belongs, both from the context (preceding/following sign) and from the sign itself.” This is a cool idea! A quick question though: if the vector model considers the context of each character, isn’t it inherently biased toward a separation of the three scripts? Since only scripts from the same category will occur together?

Indeed. You are right. However, we observe a similar behavior for both the contextual and non contextual versions of the model, suggesting that the separation of the subcorpora is inevitable, since the signs belonging to them differ in terms of shape. In addition to that, our approach is to leverage the separation of the scripts, since reducing it with no prior knowledge about the nature of the script proved challenging.

- P6: for sign2vec, did you consider different window size and type? E.g., three surrounding characters instead of one? Or only considering the words before/after rather than symmetric context? What is the dimension of the output vector? E.g., 50, 100, 500? Sorry if it is already written somewhere and I missed it.

The context size has been set to three because for CM we are able to determine sequence boundaries, since sequence dividers are used to separate them. From this information we can conclude that most words have a length of three signs or less. In addition to that, by using a window size of one, we can avoid crossing the sequence boundary, since we can only consider the word separator preceding/following the sign.

With the revised version of Sign2Vec$_d$, we aim to reconstruct signs that are often sequence initial and word final, therefore using a symmetric context was considered more appropriate, as it allows us to learn that the signs surrounding a sequence are initial and final, respectively.

The dimension of the output vector is 128, as outlined in the supplementary material.

- P6 Table 1: I understand that the authors are considering the Rand index, which is easily affected by the size of different clusters between the predicted and the actual data. Maybe the adjusted Rand index could be considered? Plus, the definition of the metrics listed in Table 1 could be explained. If the journal was a CS or CL journal that might not be necessary, but since PLOS has a larger audience, I suggest to add some brief explanations about those metrics.

We did indeed use adjusted rand index (and adjusted mutual information) but that might not have been clear. The clustering performance has been dropped from the revised version of the manuscript, so the discussion of the metrics is not relevant anymore.

- P6 "The scores were not so high because…" If I understand correctly the flow of this section, the authors wanted to validate the model on the Japanese writing system. If the results are not conclusive for Japanese, how do the authors show that the model is reliable?

We realised the impact of this, and agree with you wholeheartedly. In the revised version of the manuscript, the evaluation using Hiragana has indeed been removed. There are multiple reasons for this choice: on one hand, the overall quality of the ancient Hiragana dataset was not comparable with the Cypro Minoan one. Many signs were improperly cropped, some portions of text had many stains on the paper, etc. We therefore decided to stop using Hiragana. Another aspect regards the fact that, since our method uses the paleographic vector as its main method to find mergers between allographs in the two subcorpora, it makes little sense to use clustering as a validation step. We therefore decided to limit our validation to the application of the paloegraphic vector to 32 consensual signs in CM.

- P8 "To evaluate our model, we could only use as ground truth a set of 37 signs" I might be a bit confused here. If only those signs were used to evaluate the model, why include the other signs? This is probably already written somewhere in the text but I might have missed it.

The other signs were used during training because we were interested in evaluating the performance of the same model that we used for the application of the paleographic vector.

- P9 "This demonstrates that, while the vector is not 100% accurate, it is still a reliable method to test the hypothesis that some signs allegedly exclusive to CM1 and CM2 are in reality paleographic variants." Would it be possible to compare the accuracy obtained here with a random/majority baseline to be able to assess how high or low is the accuracy?

We now provide a more detailed statistical analysis of the results obtained from the paleographic vector, showing that obtaining our results by random chance would be highly improbable in all situations, even when considering only three signs in the case of the “Type 2” tests. We also now use the binomial distributions to calculate the probability to obtain our results to give the estimate of a random baseline.

- P11 “These results strengthen the hypothesis that the division of CM in three sub-scripts is invalid, as previously put forward on the basis of paleographic and structural evidence. The implications are of paramount importance for the script,” AFAIU, since the results do not provide a clear-cut (e.g., the accuracy of the models is not very high), I suggest that the authors could be a bit more modest when mentioning the impact of the results. The limitations of the study should also be mentioned somewhere in the conclusion, e.g., the distribution of data? The accuracy of the models and its implication on the interpretability of the results, etc…

By comparing the results obtained from both tests when compared to a random baseline, we now can show that the results we obtained cannot be due to mere chance or accident. This implies that the majority of signs exclusive to each script CAN indeed be merged with variants, and that this can be achieved via computational methods. This does, to all intents and purposes, strengthen the hypothesis that the division into three sub-systems is at best shaky.

Minor comments:

- P1, abstract: “assess if it holds up against a multi-pronged, multi-disciplinary attack”, I suggest to avoid using too strong terms such as 'attack'. However, that might be a personal preference.

We changed the term.

- P1: If space allows it, a map showing the location of the sites where the inscriptions have been found could be helpful for readers not familiar with the topic.

We have now added a map and statistics about the number of signs attested at each location.

- P4: “Almost all neural systems treating images in some ways are based on CNN, thus they seemed most fitting to our ends.” While I agree with the authors, a few references here would be nice to support this statement.

We now provide a reference to a recent survey paper, which examines the state of the art in supervised, semisupervised and unsupervised learning on images, showing the prominence of convolutional models in the field.

- P5 “we applied some quantitative measures using the MNIST dataset confirming” What are those measures again? I might have missed it.

As mentioned above, we chose to remove all discussion of our preliminary experiments, as they are not relevant for the paper and they were obtained on different datasets.

- P5: I suggest avoiding sentences such as “as mentioned above” in the paper, if you do, please refer to the exact location/section in the text.

We have now removed most such cases. Unfortunately PlosONE does not use section numbers, so using precise references would be cumbersome.

- P6: Finally, we combine the DeepCluster-v2 loss, … this is a bit abstract to follow IMHO. Maybe a toy example would help?

The loss has changed and the description has too. Hopefully it will be clearer now.

- Figure 6 and Figure 7 are hard to interpret visually. Maybe replacing the characters with points and using shapes/colors to distinguish the characters would make it easier to read?

We have debated over this, and thank you for the suggestion, but we chose not to intervene, as using images is useful and standard practice in paleography. In addition to that, at the link provided with the paper, a live version of the 3d scatterplot is available and the user can choose between visualizing images and labels, in addition to highlighting sign categories with colors.

- The format of the refs should be synchronized, for example: [2,15]: The page number seems to be missing, [10,11,30]: The publisher is missing. If the place is required as in [39], it should be added for the other references too.

The inconsistencies should have been resolved now.

Reviewer #2: The central question posed in this paper is an old one, and there seems to me some potential to try to address it with new methods of the sort proposed. However, I have serious misgivings about the way in which this research has been conducted. I hope that my specific comments below will demonstrate the grounds for my misgivings, and the reasons why on balance I felt compelled to record that the data (or rather the way in which the data were analysed) do not appear to support the conclusions offered. Unless the authors can address these issues seriously, I fear that the paper comes across as a superficial ‘confirmation’ of

pre-existing theories that may otherwise be quite adequately argued via other methods.

P1: The summary of CM inscriptions overlooks at least one further inscription from Tiryns, on the handle of a clay vessel, published by Brent Davis – this work is even on the bibliography (no. 20)! There is also a new potmark from the same site which I believe will be published by the same author.

We understand that the doubt could arise: there are three inscriptions from Tiryns, not "one" as reported in the first section by lapsus (and now correct). Nevertheless, the clay vessel inscription is actually in our dataset (see Table S1 of the Supplementary Materials) as ADD##246. TIRY Avas 002 (with reference to Davis, Maran & Wirghová 2014). As far as the new potmark is concerned, the reviewer probably refers to the re-publishing of a two-sign inscription originally published by Olivier in 1988 and Hirschfeld in 1999 as a mark. We chose deliberately not to include it until the new publication appears, and since it is only two signs long (= less than 0.1% of our dataset), this exclusion doesn't affect the results in any significant way.

P3 L64: It seems misrepresentative to say that signs not attested in the tiny repertoire of CM3 were 'allegedly discarded for linguistic reasons' (L65). Masson and Olivier both seem to have accepted that there could be signs that simply have yet to be attested in the corpus from Ras Shamra.

Indeed, Olivier treated CM3 differently to an extent. Thus, our statement refers mainly to the categorization as formulated in É. Masson (1974, p. 16), before Olivier published his corpus and reduced the signary: "Quelques signes de forme inconnue [in CM1 and CM2], nos 3, 40, 58, 71, 94, 100 et 105, indiqueraient que ce syllabaire a suivi une évolution indépendante et qu'il à pu créer des types nouveaux, dus probablement à la nécessité d'exprimer quelques termes ou noms étrangers dont l'emprunt était nécessaire dans le milieu d'Ougarit." Cf. also p. 36 on one of the Ugarit tablets: "Il rest enfin des signes au dessin nouveau, qui ont joué un rôle décisif pour nous montrer que cette écriture représente un système graphique à part … le CM 3. Ces signes ne sont pas très nombreux (… nos. 40, 94, 100 et 105), mais leur attestation … indique que leur absence dan les autres syllabaires … témoigne d'une évolution autonome de cette branche des écritures chypro-minoennes." Yet notice that Olivier had a similar methodological stance, e.g. in Olivier, J.-P. 2013, in the volume P. Steele, *Syllabic Writing on Cyprus and Its Context*: signs perceived as particular to one of the three sub-groups, or so far attested only in it, were interpreted by him as "new" and therefore computed as innovations of that script. By logic, this opposes to signs considered by Olivier as "shared" and therefore interpreted as inherited from CM1. In short,

if the interpretation of É. Masson was that signs were present/added or absent/discarded from hypothetical derivative scripts CM2 and CM3 based on the necessities of the equally hypothetical target languages, then the statement seems fair.

It is also worth noting that Olivier was openly sceptical of any linguistic distinction for CM3, making clear in Olivier 2007 that the designation is nothing more than geographical, and often using scare quotes for it (‘CM3’) – even though he maintained Masson’s categorisation.

Yes, this is true. This is already indicated in the manuscript: “(although Olivier later redefined CM3 on a geographical basis as the whole set of inscriptions from Syria).” See p. 3, lines 59-60.

P4 L106-8: The possibility that some inscriptions at the end of the chronological timespan for Cypro-Minoan might actually be written in the Cypro-Greek syllabary is raised here without any critical commentary on the implications of such an assumption. These documents could be excluded on chronological grounds, but the authors should ideally take some position on their epigraphic status (whether agnostic or not). There have been several recent discussions of the problem, including e.g.:

Duhoux, Y. (2012) ‘The most ancient Cypriot text written in Greek: The Opheltas’ spit’, Kadmos 51, 71-91.

Egetmeyer, M. (2013) ‘From the Cypro-Minoan to the Cypro-Greek syllabaries: linguistic remarks on the script reform’ in Steele, P.M. (ed.) Syllabic Writing on Cyprus and its Context, Cambridge 2012, 107-131.

Egetmeyer, M. (2017) ‘Script and language on Cyprus during the Geometric Period: An overview on the occasion of two new inscriptions’ in Steele, P.M. (ed.) Understanding Relations Between Scripts: The Aegean Writing Systems, Oxford, 108-201.

Steele, P. (2018) Writing and Society in Ancient Cyprus, Cambridge, second chapter.

To address this issue, we have edited the manuscript as follows:
- We added the methodological justification for the exclusion: inscriptions suspected of being Cypro-Greek were not included because the *Sign2Vec*$_d$ model relies on context (= distribution of signs in sequences) and is

potentially sensitive to the presence of different languages. Thus, we consider that this first machine-learning analysis should not include them.
- In the main text, we cited the bibliography containing the arguments that support the classification of the inscriptions which we considered (securely or potentially) Cypro-Greek: Egetmeyer 2010 for ##092 and Duhoux 2012, Ferrara 2012, Valério 2016 for #170-172 and ##189-190. Notice that once we consider that once the Opheltas' spi) is most probably Cypro-Greek, then all inscriptions from the same context (tomb 67 at Palaepaphos-Skales) are deemed *potentially* Cypro-Greek too.

P4 L111ff: Excluding signs on an essentially linguistic basis is methodologically worrying (the reasoning is repeated on P12). Whether or not there exist arguments in favour of linguistic differentiation, any study of sign shapes / palaeography should be blind to linguistic considerations – which surely is what the authors intend by pursuing the kinds of analysis on offer in this paper.

To be sure, the goal is to analyze Cypro-Minoan paleographically. However, in the paper we argue that a model which examines only graphic similarity leads to biased results. To counter this bias, the model was developed (in its *Sign2Vec*$_d$ version) to also consider the contexts (position in a sequence) in which signs occur. In the revised manuscript, we now provide evidence that this use of context improves results. Still, in theory the distribution of signs can be skewed in ways that affect context negatively, if a dataset includes inscriptions written in different languages.

This is the reason that we had excluded three inscriptions from Ugarit. However, we actually had data from experiments not reported here which indicated that their inclusion/exclusion does not change the results significantly. As a result, we have now included these three inscriptions in the model produced for the revised paper.

There might be some sense in excluding all the material from Ras Shamra simply on the grounds that writing practices at that site could be somewhat different from those on Cyprus – though, on the other hand, this might be a good reason for including them. But it must be all or nothing, and the methods employed here cannot seriously investigate the possibility or otherwise that CM3 should be considered as a separate entity from the rest of the CM corpus if tablets #212 and #215 are excluded (and along with them, six sign shapes thus not represented among the data used for this study).

Indeed, we did not mean to make a case for exclusion on the basis of different writing practices, as our goal is precisely to address, with our method, whether these are tied to palaeographic variation.

P4 L124ff: Given the aim to achieve more neutral analysis of palaeographic variation in Cypro-Minoan, it is a shame that the authors used published drawings, presumably largely from Olivier where some examples could be criticised as to their representation of features. Those drawings also tend to flatten some kinds of variation owing to palaeographic factors, such as the comparative width of strokes*. Perhaps it is impossible for the present study, but the results of ongoing scanning projects could be particularly beneficial to this kind of analysis because of their more accurate measurement of sign features. There is nevertheless a risk here that the results of the analysis will be affected by pre-existing assumptions and biases on the part of the person who drew the signs, given that any drawing is already in itself interpretive.

*Considerations of this kind indeed seem to have affected the analysis given the divergent clustering of signs on clay documents and signs on other supports, as noted by the authors at P8-9.

It is perhaps important to point out that:

- **Photographs of signs could not be used with our method without raising serious problems. For example, (1) several inscriptions lack a published photograph, or the photograph has insufficient resolution; (2) the model might cluster signs according to non-relevant factors, such as the color tone of the photographs.**
- **We had to rely on published drawings precisely because there are no alternative drawings.**
- **Despite a recent publication that announces the 3D modelling of a significant part of the Cypro-Minoan corpus (DOI 10.1145/3465334), the resulting dataset has not yet been published. Until new autopsies are conducted and/or new digital editions of CM become available, it is not possible to produce revised 2D illustrations of the kind this method requires.**

We share the solid misgivings of the Reviewer as to the shortcoming of *some* of the drawings published in the corpus of Olivier. Yet, as they point out, this is what is currently available to us, at least until the results of ongoing scanning projects become publicly available. Indeed, these drawings are what all Cypro-Minoan specialists (some of us co-authors and the reviewer included) use in their studies on the script. It is a standard procedure in the field of epigraphy and paleography to use published editions of texts, as most scholars do not have direct access to

the material. Even scholars who deciphered writing systems throughout history have worked with illustrations.

In any event, there is one remarkable piece of evidence which suggests the drawings do not affect results negatively: our model was able to accurately predict that sign 087 on inscription ##063. ENKO Abou 060 is to be corrected to 088 (Table S2), even though the drawing by Olivier (which is what we used) does not represent the stroke that is the main distinguishing feature between the two signs, and which is observable only on photographs.

P9 L316-8: "This property supports the argument that CM2 is not a script distinct from CM1, but rather a form of the same writing system that differs mainly due to the use of a different writing medium as well as scribal style (smaller and more angular signs)."

This seems to me to be quite a bold claim (not that CM2 is not a separate script in its own right, which is surely at some level true, but that the present investigation can be used as evidence for such a position). I am not convinced that the results can only be read in this way. For one thing, it may be that the quite consistent way in which CM2 signs were drawn (presumably by Olivier?) predisposed them to a differential analysis by the neural network – as I mentioned above, this is a serious risk to the results of the study and needs to be considered carefully.

It is hard to prove that the idiosyncrasies of one hand (the clay tablets were drawn by É. Masson 1974, 1978, 1989, not Olivier) do not affect the results significantly. To test this, one would need multiple datasets with drawings of the same CM inscriptions by different individuals, which do not exist (incidentally, note that even the published corpus of Olivier 2007 contains drawings by multiple hands). However, the following is even more informative: É. Masson drew many other inscriptions (on different media) used in Olivier 2007 and in our dataset, and yet it is only the clay tablets that are clearly set apart in the model.

It would also be helpful to know to what extent differences of scale have been factored in. The signs of the CM2 tablets are far smaller than signs on many other supports, and this makes a difference a) to what it was possible for the author to render, and b) to the accuracy of any modern drawing of the signs. Published editions tend to flatten the degree of difference in size between signs in different inscriptions, but this could indeed be a significant factor in their recognisability (whether to ancient humans or modern computational methods).

The images in our dataset do not change the proportion of the original drawings, so they carry the same degree of normalization—if there is

any—seen on the latter. Because of the characteristics of the existing published corpora, it is difficult (if not impossible) to factor in scale in a consistent way, so our study focuses on shape independently from size. We agree that the differences in size (on the actual inscriptions) affect the recognizability of signs, but this is because size influenced shape (meaning the way the sign was drawn) in the first place. The model provides evidence that its results contain no significant scale-induced bias: the well-accepted CM2 variants of certain signs occur separately from their CM1 variants in the vector space of our model. (This spatial distance reflects the differences in shape between the two forms, which in turn can be due to differences in size and other paleographical factors, as pointed out by the Reviewer.) Yet, at the same time, variants of the same sign occur along the same axes. In short: what size affects directly is shape, and shape is precisely what the model addresses.

P10-11: In the section 'Application of the Vector', it is clear that the authors seem to have drawn conclusions that supported pre-held beliefs, but very little information is given as to how the conclusions are supported. Accuracy levels such as 6/10, 7/10, 2/3, 3/3 need to be explained in some detail – what exactly is denoted by these numbers, and what does 'accuracy' mean here? Have the results been tested for statistical significance?

- "8/13 accuracy at one" means that the vector provided 8 correct predictions out of 13 tested hypothesis, considering only the closest sign (cf. Table S5).

- "9/13 accuracy at two" means that the vector provided 9 correct predictions out of 13 tested hypotheses, considering the two closest signs (cf. Table S5).

In any event, we have edited the text to make this point clearer.

P12 L449-451: "If the inscriptions in our dataset (mainly CM1 and CM2) represent the same script, then the likelihood increases that this single script recorded the same language."

This is an extremely bold and methodologically unsound claim. There are countless examples across the world and across different time periods of different languages being written in a single script / writing system. The language-related considerations offered here do not seem appropriate to the purposes of the paper.

This statement derived from our observation of the general results (as Sign2vec$_d$ uses context), but also from the fact that including or excluding

CM3 inscriptions containing shapes exclusive of CM3 in our original simulations did not affect the results significantly. Moreover, we spoke of an increasing likelihood, not proof. But we agree with the Reviewer that this needed addressing, so we have amended this statement in the revised manuscript to keep with conclusions that are germane to the study at hand and its specific results.
